# Altered neural electrophysiological properties in the anterior cingulate cortex in a mouse model of Prader‐Willi syndrome

**DOI:** 10.1113/JP290255

**Published:** 2026-08-14

**Authors:** Volodymyr Rybalchenko, Ryan Butler

**Affiliations:** ^1^ Department of Psychiatry University of Texas Southwestern Medical Center Dallas Texas USA; ^2^ Department of Pediatrics University of Texas Southwestern Medical Center Dallas Texas USA; ^3^ O'Donnell Brain Institute University of Texas Southwestern Medical Center Dallas Texas USA

**Keywords:** anterior cingulate cortex, hedonic feeding behaviour, ion channel expression, neuronal excitability, Prader‐Willi syndrome

## Abstract

**Abstract:**

Prader‐Willi syndrome (PWS) is a neurodevelopmental genetic disease associated with multiple metabolic and behavioural abnormalities converging into a distinctive clinical phenotype characterized by insatiable appetite leading to hyperphagia and eventual morbid obesity. The PWS spectrum results from deficiencies in paternally imprinted chromosome 15q11‐13 region clustering around non‐coding RNA multiple‐repeat gene *Snord116*. A PWS mouse model with paternal *Snord116* deletion (Snord116del) revealed multiple expected behavioural traits but failed to reproduce obesity in experimental paradigms designed to uncover homeostatic hypothalamic mechanisms of hyperphagia, while the possibility for pathologic hedonic overdrive underlying hyperphagic behaviours was not studied. In Snord116del mice, we examined functional properties of pyramidal neurons (PyNs) in the anterior cingulate cortex (ACC), the brain area commonly associated with goal‐oriented and choice‐outcome processing, including the value assessment of food items. We found indications of higher dendritic complexity and stronger afferent excitatory connectivity compared to controls. A strong excitatory input into Snord116del PyNs was balanced by a more hyperpolarized resting membrane potential, rendering lower soma excitability, improved signal‐to‐noise discrimination and stronger low‐pass filtering. The enhanced excitatory network‐tuning ability originating from *Snord116* deficiency may explain the previously reported better performance of Snord116del over wild‐type mice in working‐for‐food behavioural tests, whereas in humans it might entail exaggerated reward‐seeking behaviour since early childhood when food is the main attractant. Our analysis of previously published genomic databases revealed candidate genes responsible for the abnormal functional neuronal phenotype caused by Snord116 deletion, including K^+^ and Na^+^ voltage‐dependent ion channels, protein kinases, phosphatases and components of the mechanistic target of rapamycin (mTOR) intracellular signalling pathway.

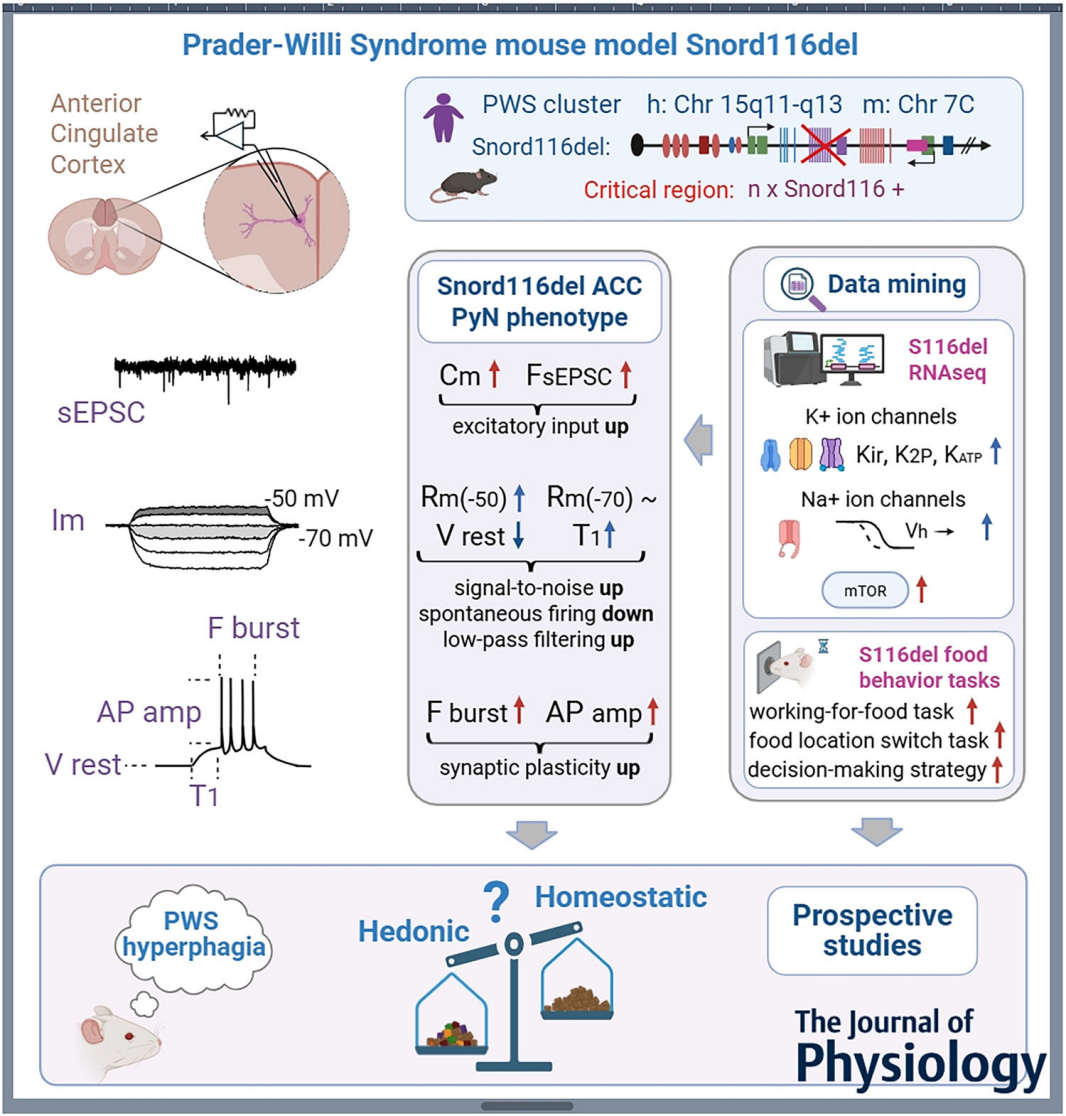

**Key points:**

- Altered biophysical characteristics and parameters of neuronal connectivity in pyramidal neurons in the anterior cingulate cortex (ACC) in *Snord116* deletion mice.
- Alterations include augmented afferent synaptic input, altered resting state and firing properties of ACC pyramidal neurons.
- Our findings uncover a possible mechanistic basis for altered ACC functionality in Prader‐Willi syndrome.

## Graphical abstract

**Abstract figure legend** Prader‐Willi syndrome (PWS) originates from deficiencies in chromosome 15q11‐13 region clustering around a critical region containing multiple‐repeat non‐coding RNA gene *Snord116*, resulting in metabolic and behavioural abnormalities leading to hyperphagia and obesity. In the PWS mouse model *Snord116del*, we examined functional properties of pyramidal neurons (PyNs) in the anterior cingulate cortex (ACC) commonly associated with goal‐oriented processing, including the value assessment of food items. We found that multiple electrophysiological parameters characterizing passive and active properties were altered in *Snord116del* mouse neurons in a way to emphasize increased excitatory input, synaptic plasticity and signal‐to‐noise efficiency, while reducing spontaneous firing. Our analysis of genomic databases revealed multiple ion channel and mechanistic target of rapamycin (mTOR) signalling protein alterations potentially contributing to Snord116del neuronal phenotype, combined with published evidence of stronger acuity of Snord116del mice in food foraging behavioural tasks. Our work prompts prospective studies on whether PWS *o*riginates from insufficiently studied hedonic food drive rather than homeostatic perturbations.

## Introduction

Prader‐Willi syndrome (PWS) is a rare genetic disease predominantly caused by variable‐length mutations or deletions of the paternal copy or maternal uniparental disomy of imprinted genes located along the chromosome 15q11‐q13 region. It is characterized by early neonatal hypotonia and feeding problems, but during late childhood and into adulthood, a symptomatic lack of satiety and poor control of feeding behaviour occur in a significant proportion of patients, leading to hyperphagia and often extreme obesity (Kummerfeld et al., 2021; Mendiola & LaSalle, 2021). Common features also include hypogonadism, elevated levels of ghrelin and reduced levels of growth hormone (GH) (Tauber et al., 2019), indicating disruptions in endocrine regulation. The spectrum of the behavioural traits and mental conditions reported in PWS included psychosis, depression, anxiety, obsessiveness and compulsiveness, which can result from a confounding combination of genetic predisposition and social environment (Whittington & Holland, 2018).

The imprinted chromosome 15q11‐q13 region contains coding, non‐coding and imprinting control centres highly conserved in mammalian species, with orthologous murine genes harboured on chromosome 7C. The multiplicity of clinical studies revealed that the disruption of the minimal critical region narrowly restricted around the multiple‐repeat (∼30 copies) cluster of the non‐protein coding small C/D box nucleolar RNA *Snord116* recapitulates most of the symptoms of the PWS described for longer *Snord116*‐encompassing dysfunctional clusters, suggesting that the disruption of *Snord116* plays a fundamental role in PWS pathogenesis (Cavaille, 2017). Two different mouse models (PWScr, *Snord116del*) with paternal *Snord116* deletion were developed (Ding et al., 2008; Skryabin et al., 2007) and subsequently used to study the neuronal origin of PWS‐related disturbances, relying on findings that *Snord116* in rodents is expressed exclusively in the brain (Rozhdestvensky et al., 2016). Behaviour results varied between studies, with *Snord116del* mice generally demonstrating a subtle phenotype with a reduced anxiety in open field tests but increased in elevated plus maze, no defects in working and spatial memory, but some anomalies in cued fear conditioning and decreased startle response associated in humans with either a depressive or an opposite anxiolytic state (Adhikari et al., 2019; Ding et al., 2008; Lassi, Maggi et al., 2016; Scott et al., 2025; Zahova et al., 2021; Zieba et al., 2015). These *Snord116del* behavioural phenotypes convey strong face validity to human PWS pathology. Notably *Snord116del* mice performed better than controls in working‐for‐food and food location switching tasks due to better timing strategies and decision making (Lassi, Maggi et al., 2016).

Many studies using *Snord116del* mouse models were focused on hypothalamic mechanisms, examining homeostatic feeding behaviour, aspects of sleep and molecular prohormone processing – all of which have been reported to be dysregulated in PWS patients. Inconsistent results were obtained on whether the expression of the regulatory and coding genes for prohormone convertase PC1 (*Nhlh2* and *Pcsk1*) is downregulated in *Snord116del* hypothalamic neurons (Burnett, LeDuc et al., 2017; Polex‐Wolf et al., 2018). A separate study using selective deletion of *Snord116* in NPY‐expressing orexigenic neurons produced a mouse feeding phenotype identical to that with global *Snord116* deletion (Qi et al., 2016), but later studies reported no changes in NPY and other leptin/melanocortin pathway genes (*Pomc, Lepr, Agrp*) in adult mice with targeted hypothalamic *Snord116* deletion (Polex‐Wolf et al., 2018), providing further uncertainty about the actual role of disrupted hypothalamic genes in regulating the feeding behaviour of *Snord116del* mice. A recent study linked *Snord116* deficiency in the lateral hypothalamus with partial loss of orexin neurons, rapid eye movement (REM) sleep increase and decreased homeostatic response following sleep deprivation, although no changes in food‐seeking behaviour were observed in this hypothalamic study (Pace, Falappa et al., 2020). This was in line with multiple reports that *Snord116del* mice do not produce an overweight phenotype in standard feeding protocols, and only mild hyperphagia was observed in some studies if smaller weights of *Snord116del* mice originating from GH deficiency were used as norm factors (Burnett, LeDuc et al., 2017; Ding et al., 2008; Lin et al., 2014; Qi et al., 2017). Moreover, *Snord116del* mice demonstrate weight‐gain resistance after a switch to a high‐fat diet (HFD) (Ding et al., 2008; Qi et al., 2016). The inability to establish marked hyperphagia and obesity in *Snord116del* mice in standard experimental protocols designed to examine homeostatic feeding behaviour leaves a possibility that human obesity in PWS may have a predominant hedonic component driving poorly controlled feeding behaviour of the cortical/mesolimbic origin. The experimental approaches specifically designed to explore hedonic feeding behaviour in *Snord116del* mice have not been previously applied systematically despite certain indications that *Snord116* deletion can alter the functional state of cortical networks in isolation from thalamic inputs. Namely, the power of carbachol‐stimulated theta waves measured with multielectrode arrays in mouse cortical primary neuron cultures was significantly higher in *Snord116del* preparations compared with controls, and single‐unit recorded neuronal spiking was desynchronized and significantly delayed upon switching bursting patterns in *Snord116del* neurons (Pace, Colombi et al., 2020). We hypothesized that neuronal abnormalities may also take place in *Snord116del* mouse cortical areas involved in decision making and reward seeking that ultimately control hedonic feeding behaviour. To test this hypothesis we examined multiple electrophysiological parameters of pyramidal neurons in *ex vivo* brain slice preparations of the anterior cingulate cortex (ACC) in *Snord116del vs*. control mice. We found significant differences indicative of altered neuronal morphological and intrinsic excitability properties originating from *Snord116* deficiency, with high potential for disturbing global cortical network integrations in this brain area. We analysed several previously published transcriptomic databases obtained in PWS human subjects and *Snord116del* mice and identified multiple groups of differentially expressed genes encoding intracellular signalling cascade molecules and voltage‐dependent ion channels, whose disruption may result in abnormal neuronal phenotypes in cortical neurons in *Snord116del* mice revealed through our study, and may potentially contribute to elevated hedonic feeding drive in PWS patients.

## Methods

### Ethical approval

All animal mouse studies contributing to this work were approved by the Institutional Animal Care and Use Committee at the University of Texas Southwestern Medical Center at Dallas (animal protocol number: 2018‐102619‐G) and were conducted in compliance with animal ethical principles under which the *Journal of Physiology* operates. UT Southwestern is AAALAC accredited and has a federally assured animal care programme with OLAW/PHS Animal Welfare Assurance Number D16‐00296 (A3472‐01) and fully adheres to the National Institutes of Health (NIH) and United States Department of Agriculture (USDA) regulations for humane care and use of laboratory animals (USDA/APHIS Number 74‐R‐0072).

### Animals

We used the C57Bl/6N Snord116(p−/m+) mice (B6.Cg‐Snord116tm1.1Uta/J, stock 008149) originating from The Jackson Laboratories, backcrossed onto a C57Bl/6N background over 10 generations (Osborne‐Lawrence et al., 2023), obtained from Dr. Zigman Lab in the UT Southwestern Medical Centre. Experimental Snord116(p−/m+) mice, which carry a paternally inherited chromosome 7 lacking the Snord116 gene cluster (called further Snord116del mice) and control Snord116(p+/m+) (called WT) littermates, were generated by crossing male C57Bl/6N Snord116del mice with female C57BL/6N mice. Housing conditions included a 12‐h light–dark cycle and *ad libitum* access to standard chow and water. Adult mice aged 21‐ to 41‐week‐old of both sexes were included in this study.

### Genotyping

Genotyping was performed using genomic DNA extracted from tail snips and three primers to detect the *Snord116del* and WT Snord116 alleles: Snord116‐M634 (5′‐TGGATCTCTCCTTGCTTGTTTTCTC‐3′), Snord116‐M635 (5′‐AATCCCCAACCTACTTCAAACAGTC‐3′) and Snord116‐M636 (5′‐TTTACGGTACATGACAGCACTCAAG‐3′). The following PCR protocol with an annealing temperature at + 64°C generated a WT band of 435 bp and a mutant band of 337 bp.

### *Ex vivo* brain slice preparation

The mouse was removed from the transport box and placed for acclimation into a custom‐made rectangular plastic box (15 × 10 × 10 cm), in which a narrow bottom chamber was separated from the upper chamber by a gas‐permeable false‐bottom platform covered with a cloth napkin and nest‐building material. The box was equipped with the anaesthetic‐delivery tube connecting the bottom chamber with the exterior orifice allowing the anaesthetic infusion into the bottom chamber without disturbing a (usually sleeping after the initial acclimation) mouse in the upper chamber. The box had a few ventilation holes in the side wall for undisturbed breathing during acclimation. The acclimation box was covered with a paper towel to prevent the animal from having any visual stimulation, and the mouse remained undisturbed for 30–60 min. Then ∼1 ml of isoflurane was infused into the bottom evaporation chamber without opening a box and disturbing the animal. The animal did not come into direct contact with isoflurane at any point during the procedure. After 10–15 s the mouse was removed from the box, the absence of the foot‐pinching reflex was verified and mouse was quickly decapitated using surgical scissors. The whole brain was quickly extracted and used for preparing coronal slices. The cortical section of the brain was extracted and transferred to the slicing chamber of the vibratome (LEICA VT1200s, Nussloch, Germany) filled with ice‐cold dissection buffer containing (mM): NaCl 84, KCl 3, NaH_2_PO_4_ 1.25, CaCl_2_ 0.5, MgSO_4_ 7, NaHCO_3_ 26, glucose 20, sucrose 70, ascorbic acid 1.3, kynurenic acid 2 (brought to pH 7.3 by continuous saturation with carbogen gas containing 95% of O_2_ and 5% of CO_2_). Coronal brain slices (300 µm thick) containing the ACC area were cut out and transferred to equilibrate in a storage chamber filled with artificial cerebrospinal fluid (ACSF) solution containing (mM): NaCl 125, KCl 5, NaH_2_PO_4_ 1.25, CaCl_2_ 2, MgCl_2_ 1.3, NaHCO_3_ 25, glucose 12, continuously saturated with carbogen gas to keep pH 7.3. Slices were maintained in a storage chamber at 34°C before being used in experimental tests 1 to 4 h after dissection.

### Whole‐cell patch‐clamp recordings

Slices were placed in a temperature‐controlled submerged recording chamber (AutoMate Scientific, Berkeley, CA, USA) custom‐adjusted to reduce volume (∼400 µl) and to provide a laminar flow aimed at minimizing alkaline pH drift due to CO_2_ loss at the surface. The main extracellular solution (ACSF, same as in the storage chamber) in the 60‐ml storage container was continuously heated to 34°C, saturated with carbogen gas and circulated at 2 ml/min between the container and the recording chamber passing through CO_2_‐impermeable Teflon tubing and the inline heater (Warner Instruments, Hamden, CT, USA), keeping the temperature of 37°C in the recording chamber. Pyramidal neurons (PyNs) in the ACC layer 2/3 were visually identified with an upright microscope (Olympus BX51 WI, Hachiojy, Tokyo, Japan) equipped with an infra‐red light sensitive camera (Prime BSI Express, Teledyne DALSA, Waterloo, Canada) and patch‐clamped using borosilicate glass (BF150‐86‐10, Sutter Instruments, Novato, CA, USA) patch pipettes pulled with a pipette puller (P‐1000, Sutter Instruments) to form open pipette resistance ∼ 3–5 MΩ while filled with the intracellular solution containing (mM): K‐methanesulfonate 130, NaCl 5, MgCl_2_ 2, EPES 10, EGTA 1, MgATP 2, Na_2_GTP 0.2 (pH∼7.3 adjusted with NaOH). Immediately after the membrane patch rupture, the passive neuron membrane parameters (membrane capacitance *C*_m_ and input resistance Rm) were measured while in the voltage‐clamp mode. Typical membrane access series resistance (Rs) ranged between 15 and 30 MOhm, and few cells were accepted with Rs∼35 MOhm (see Table S1, Data Inclusion), as the possible skewing effect of Rs on recorded parameters was minimized by routinely engaging a standard Rs electronic compensation feedback circuitry embedded in the recording software interface. Rs was monitored periodically during experiment, and data were accepted if Rs did not deviate from the initial value by 25%. Naturally occurring resting membrane potential (*V*_rest_) was measured in the current‐clamp mode (with no current injected) within the first 2 min of recordings. *V*_rest_ usually remained stable (±2 mV) during the experiment but could transiently become more hyperpolarized after a series of action potential (AP) trains evoked by depolarizing current injections. Neurons with *V*_rest_ more positive than −50 mV were routinely discarded, except three cells with *V*_rest_ between −49 and −45 mV (Table S1, Data Inclusion), for which normalized membrane conductance (*G*_m_/*C*_m_) was within 0.4*SD from mean values for their genotype group, meaning that depolarized subthreshold resting‐state occurred naturally, and not as a result of poor seal or leaky membrane. The identity of each pyramidal neuron was verified by recording a specific pattern of trains of APs distinct from fast‐spiking activity of interneurons.

All recordings were performed using Multiclamp 700A amplifier, Digidata 1440A digitizer and pClamp 10.2 software (all Molecular Devices, San Jose, CA, USA). Electrical signal was sampled at 5 kHz and low‐pass filtered at 1 KHz with an eight‐pole low‐pass Bessel filter using Multiclamp/pClamp functionality. Spontaneous excitatory postsynaptic currents (sEPSCs) were typically recorded at the beginning of the experiment (after the initial intracellular solution equilibration of ∼10 min) to avoid possible distal dendrite morphological deformities by slowly developing osmotic effects. The pipette solution composition was designed to keep the Nernst equilibrium potential for Cl^−^ anions at ∼ −70 mV. We recorded sEPSCs at a holding membrane potential (*V*_hold_) of −70 mV in voltage‐clamp mode, which efficiently nullified GABA‐A receptor mediated Cl^−^ currents during recordings (verified by the total disappearance of any miniature unitary currents in presence of 20 µM AMPA receptor blocker 6‐cyano‐7‐nitroquinoxaline‐2,3‐dione (CNQX) in control experiments). We purposely did not block GABA‐A receptors during sEPSC recordings, because after switching to the current‐clamp mode, we intended to keep the neuron in the most natural environmental state with unchanged *V*_rest_ and AP firing patterns. Blocking the tonic contribution of background GABA‐A receptor synaptic inputs would inevitably skew both measured metrics. sEPSC frequency and amplitude were calculated from contiguous 2‐min recordings using the miniature‐spike template search and analysis algorithm embedded into the pClamp 10.2. A search algorithm was trained on real sEPSC spikes; it combined five different templates representing elementary miniature events with different kinetical properties and was applied to all analysed sEPSC recordings with no intermittent modifications. To measure electrical membrane (chord) conductance (*G*_m_/*C*_m_) at various subthreshold membrane potentials (Fig. 3*C–G*), the 100 ms long 10 mV incremental voltage steps between −120 and −50 mV were applied from *V*_hold_ −70 mV in the voltage‐clamp mode.

Single APs (Fig. 2) were generated in the current‐clamp mode at natural *V*_rest_ by injecting brief suprathreshold 1 nA current steps of variable duration (between 0.6 and 3 ms in 0.4 ms incremental steps) until consecutive reproducible AP shapes were generated (typically at 1.4 ms). Alternatively, the series of AP trains were evoked by 50 pA incremental 500 ms current steps within the range of −200 to +500 pA starting from natural neuron resting potential (*V*_rest_) (Figs. 4, 5, 6). Numerical parameters describing the individual‐ or trains of APs were calculated semi‐automatically using the AP search and analysis algorithm embedded in the pClamp 10.2. To obtain and calculate the rheobase values (*I*_rhe_, Fig. 3*A,B*), no specific experimental protocol was applied during the experiment due to rational consideration that typical *I*_rhe_ protocol (Δ*I* = 10pA step followed by at least 10 s interpulse interval) would take long enough time (up to 40 cycles) undermining cell stability and hampering successive application of multiple planned protocols during one experiment. Instead, chord *I*_rhe_ values were extracted from records of AP trains evoked by Δ*I* = 50pA, 500ms incremental current steps using the formula *I*_rhe_  = *I*_last_ + 10*(6 − *n*), where *I*_last_ is the last step producing no APs, and *n* is the number of APs generated upon the next resultative step (*n* = 5 was set in a few cells with more than 5 APs generated upon the first resultative step). The AP instant frequency parameter was calculated as the inverse value of the interspike interval between first and second APs Δ*t*_1_ (*F*_inst_ = 1/Δ*t*_1_). The cumulative frequency function for *n* APs in the train (Fig. 5*A1*) was calculated by pClamp 10.2 AP search algorithm that divides the number of consecutive APs minus one (i.e. the number of interspike intervals) by the total time passed between APs 1 and *n* (Δ*t*_n_): (*F*_cml_(*n*) = (*n* − 1)/Δ*t*_n_).

### Statistics

The data collection and analysis were conducted by the experimenter blinded for the animal genotype until a final disclosure and data separation into two genotype groups. A total of 28 mice were used for this study, including 13 controls and 15 *Snord116del*, among which were 14 males and 14 females. Data were collected from 63 cells recorded in 31 brain slices (∼ 2 cells per slice). Several experimental protocols (in both voltage‐ and current‐clamp modes) in various combinations were applied to each experimental cell, which explains variable number (*n*) of cells included in the statistical analysis for each particular parameter. The full list of animal genotypes, cells, sets of parameters collected from each cell and log file excerpts for each cell is included in Table S1 (Data Inclusion), and shows explicit inclusion/exclusion status for each cell in the statistical pool for each figure included in this work. The statistical analysis and non‐linear curve fitting were done using Prism 11 software (GraphPad, Boston, MA, USA). Descriptive parameters for passive neuron properties, parameters describing various phases of single APs and characteristics of sEPSCs were analysed in two alternative ways using a two‐way ANOVA to analyse the interaction between sex and genotype and statistical differences within these subdivisions, whereas a separate non‐parametric two‐tailed Mann‐Whitney (*t* test) with multiple comparisons was used to extract statistical parameters (mean, SD) for individual data points and to analyse significant differences (*P* < 0.05) between genotypes. The two‐way ANOVA analysis for all analysed sex/genotype parameters revealed no interaction or sex differences. Therefore, the experimental data for males and females were pooled for presentation in the figures. The results of statistical analysis for all parameters (including those calculated separately for males and females) are available in Table S2 (Data Statistics). The Wilcoxon matched‐pairs single rank test was used to determine the statistical significance of differences between genotypes upon analysing the sets of variable parameters represented by non‐linear functions of: (i) injected membrane current (Δ*I*_m_ steps) or (ii) a consecutive number (*n*) of the AP within a generated train of APs. All statistical results presented in the main text and figures are mean ± SD.

## Results

### *Snord116del* neurons have larger total membrane surface and are enriched in synaptic inputs

**Figure 1:**
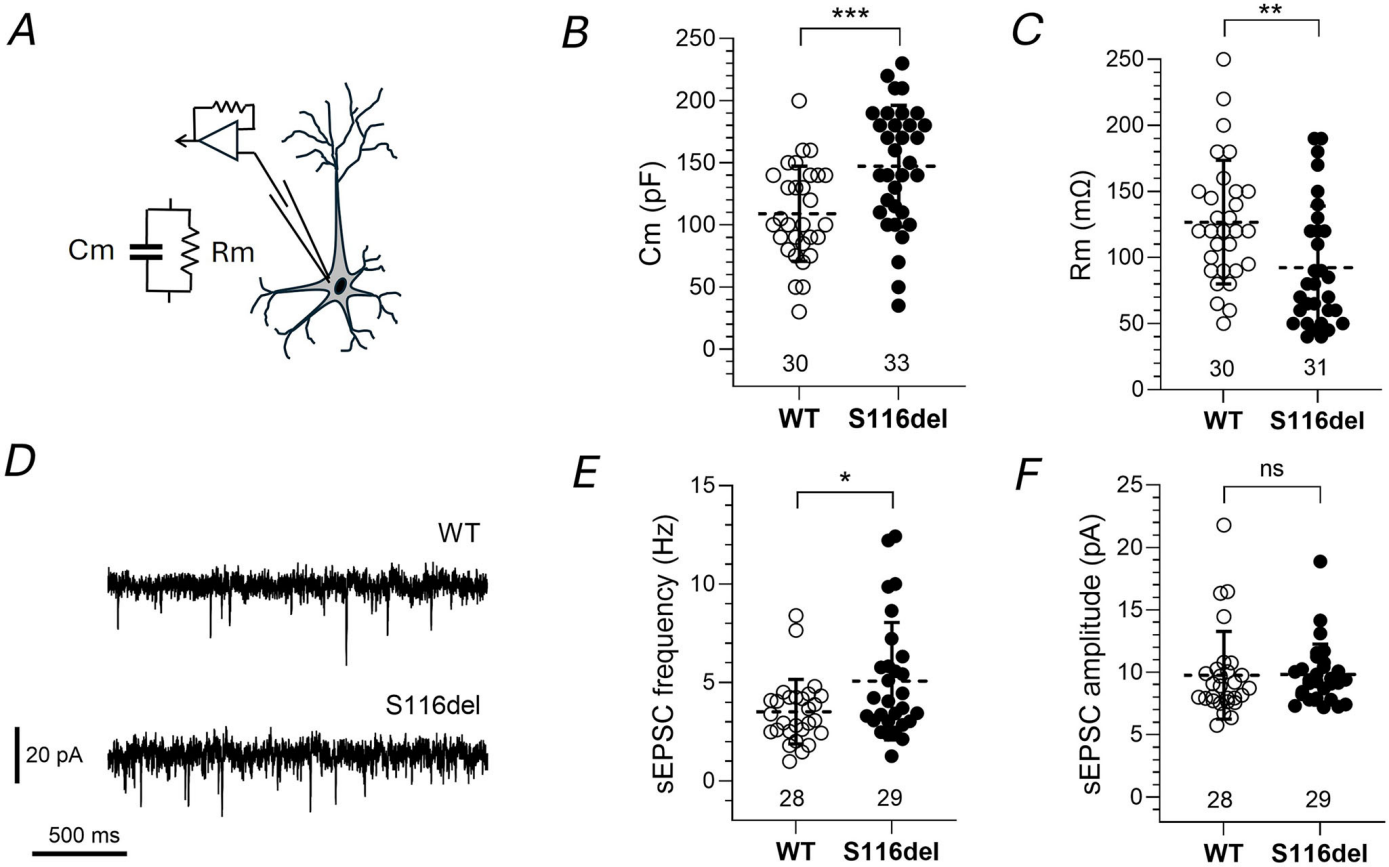
*Snord116del* neurons have a larger total membrane surface and are enriched in synaptic inputs *A*, schematic representation of the plasma membrane passive electrical parameters measured in patch‐clamped neurons. *B, C*, statistics of membrane capacitance (*B*) and input resistance (*C*) obtained in voltage‐clamped (*V*_hold_ −70 mV) anterior cingulate cortex (ACC) pyramidal neurons from control (WT) and *Snord116del* mice. *D*, fragments of recordings of spontaneous excitatory postsynaptic currents (sEPSC) in WT and *Snord116del* neurons. *E*, *F*, statistics of sEPSC frequency (*E*) and amplitude (*F*) measured in WT and *Snord116del* neurons. The number of tested cells is indicated at the bottom, mean values are represented by dashed lines on the graphs and error bars represent ± SD. *P < 0.05, **P < 0.01, ***P < 0.001 (see numerical and *P* values in the text).

Although most neuroimaging studies of PWS patients revealed normal brain morphology, some reports described cases of ventriculomegaly, reduced parietal‐occipital grey matter volume and more general cortical atrophy in patients of various ages (Manning & Holland, 2015). PWS is not classified among neurodegenerative disorders, but it is plausible that certain morphological properties of neurons may be affected by PWS‐linked mutations. Recent immunohistochemical studies in mouse PWS models indicated that *Snord116* deficiency may lead to a reduction in soma size in forebrain neurons and in cerebellar Purkinje neurons due to reduced‐size nucleoli (Burnett, Hubner et al., 2017). Rodent MRI revealed a reduced size of hippocampal grey‐matter in these mice (Lassi, Priano et al., 2016). To test whether other neuronal features might be altered in *Snord116* (p‐/m+) mouse model of PWS (called *Snord116del* in the text, and S116del in figures), we applied patch‐clamp electrophysiological techniques to measure a wide range of functional parameters of neurons in *ex‐vivo* brain slice preparations (Fig 1*A*). We were interested in examining neurons in the ACC, as this brain area in neuroimaging studies on PWS patients revealed increased activation during compulsive skin picking (Klabunde et al., 2015), increased resting state connectivity (Zhang et al., 2013) and increased glucose metabolism (Kim et al., 2006) compared to control subjects. Membrane properties of pyramidal neurons in the ACC layers 2/3 (L2/3) were measured in the voltage‐clamp mode at holding membrane potential (*V*_hold_) −70 mV. In accordance with smaller soma sizes previously reported in fixed brain preparations in *Snord116del* mice (Burnett, Hubner et al., 2017), we expected to obtain lower values for membrane capacitance (*C*_m_), a correlate of the total membrane surface. Surprisingly, we found that *C*_m_ values were significantly increased in *Snord116del* neurons (Fig. 1*B*; WT: 109 ± 38 pF (*n* = 30), *Snord116del*: 147 ± 49 pF (*n* = 33), *P = *0.0009), and accordingly the membrane input resistance (*R*_in_) was significantly lower (Fig. 1*C*; WT: 127 ± 47 MΩ (*n* = 30), *Snord116del*: 92 ± 47 MΩ (*n* = 31), *P = *0.0031), indicating a larger total membrane surface area for ACC pyramidal neurons in *Snord116del* mice. Soma and dendritic tree collectively contribute to the total surface area, with the dendritic tree contributing a significantly higher share in pyramidal neurons. It is possible that higher *C*_m_ values in *Snord116del* neurons reflect a more developed dendritic tree, and as a result a higher number of excitatory synaptic connections relative to WT neurons. This assumption could be verified by measuring spontaneous excitatory postsynaptic currents (sEPSC). We recorded sEPSCs in both types of neurons and found that sEPSC frequency is significantly higher in pyramidal neurons from *Snord116del* mice than in controls (Fig. 1*E*; WT: 3.52 ± 1.64 Hz (*n* = 28), *Snord116del*: 5.07 ± 2.98 Hz (*n* = 29), *P = *0.0450), whereas the sEPSC amplitudes were unchanged (Fig. 1*F*; WT: 9.76 ± 3.52 pA (*n* = 28), *Snord116del*: 9.82 ± 2.44 pA (*n* = 29), *P = *0.385). A similar increase in sEPSC frequency was recently reported in hippocampal and dentate gyrus neurons in mouse models of neuronal hypertrophy (mTOR gain of function), where this increase was linked to higher dendritic complexity and synaptic spine density (Cullen et al., 2023; Tariq et al., 2022). Thus, two corroborating findings of (i) increased sEPSC frequency and (ii) increased *C*_m_ in *Snord116del* ACC pyramidal neurons would indirectly indicate that *Snord116* deficiency might result in more complex dendritic tree and higher excitatory afferent synaptic connectivity compared to WT neurons.

### *Snord116del* neurons are more hyperpolarized at rest and generate higher amplitudes of action potentials compared to WT neurons

**Figure 2:**
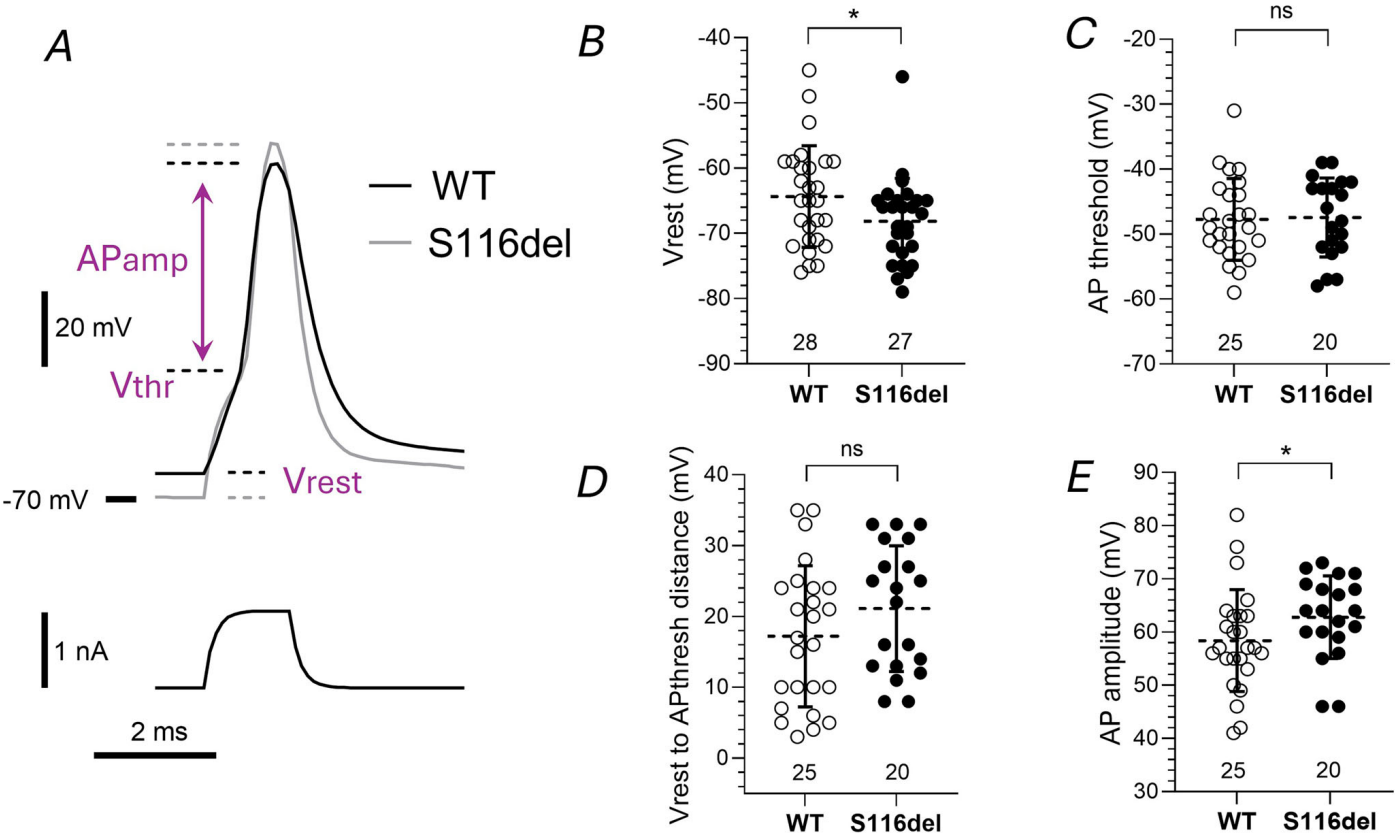
*Snord116del* neurons are more hyperpolarized at rest and fire stronger action potentials than WT neurons *A*, single action potentials (APs) evoked by a brief suprathreshold depolarizing pulse in current‐clamped representative neurons from WT and *Snord116del* mice reflecting differences in resting membrane potential (*V*_rest_) and in basic characteristics of APs. *B*–*E*, statistics of *V*_rest_ (*B*), AP threshold (AP_thr_) (*C*), voltage barrier to fire APs (*D*) and AP amplitude (AP_amp_) (*E*) in WT and *Snord116del* neurons. The number of tested cells is indicated at the bottom, mean values are represented by dashed lines on the graphs and error bars represent ± SD. *P < 0.05 (see numerical and *P* values in the text).

Elevated excitatory connectivity, as suggested by our data above, would often increase propensity for epileptiform cortical activity, which has not been reported in patients or in mouse models of PWS. We suggested that certain compensatory mechanisms may exist to balance elevated cortical excitatory connectivity in *Snord116del* mice. To test this hypothesis we examined intrinsic neuronal excitability properties in *Snord116del* and WT mice. In the current‐clamp mode of patch‐clamp recording, we measured the resting membrane potential values (*V*_rest_) immediately after membrane patch rupture, and we subsequently evoked a single action potential (AP) by brief injections of high suprathreshold current (1 nA, ∼3 ms; Fig. 2*A*). We obtained significantly more hyperpolarized *V*_rest_ values in neurons from *Snord116del* mice relative to controls (Fig. 2*B*; WT: −64.4 ± 7.8 mV (*n* = 28), *Snord116del*: −68.1 ± 6.6 mV (*n* = 27), *P = *0.0454). The membrane potential threshold for AP firing (*V*_thr_) values was not significantly different between neurons from WT and *Snord116del* mice (Fig. 2*C*; WT: −47.7 ± 6.3 mV (*n* = 25), *Snord116del*: −47.5 ± 6.1 mV (*n* = 20), *P = *0.834). The voltage barrier to firing AP (*V*_thr_ − *V*_rest_) showed a tendency to be higher for neurons in *Snord116del* than in WT mice (Fig. 2*D*; WT: 17.2 ± 10.0 mV (*n* = 25), *Snord116del*: 21.1 ± 8.9 mV (*n* = 20), *P = *0.143), suggesting a lower intrinsic neuronal excitability for ACC pyramidal neurons in *Snord116del* relative to WT mice. Both the more hyperpolarized *V*_rest_ and the higher energy barrier to fire AP would compensate for the apparent higher cortical excitatory connectivity and would provide baseline stability of the ACC cortical network in *Snord116del* mice.

The amplitude of the AP (AP_amp_) is an important parameter defining how far the somatic depolarization spike back‐propagates along the dendritic tree and forward‐propagates along the axonal tree, which includes significant unmyelinated sections in cortical pyramidal neurons, particularly in layers 2/3 (Tomassy et al., 2014; Wildenberg et al., 2020). The AP_amp_ depends inversely on the level of subthreshold steady‐state inactivation of voltage‐gated sodium channels (VGSCs) that can be as high as 60% at depolarized membrane potentials (*V*_m_) approaching *V*_thr_ (Groome et al., 2011). The AP_amp_ values of single APs illustrated in Fig. 2*A* were significantly higher in ACC neurons from *Snord116del* than in WT mice (Fig. 2*E*; WT: 58.4 ± 9.6 mV (*n* = 25), *Snord116del*: 62.8 ± 7.8 mV (*n* = 20), *P = *0.0439), in accordance with more hyperpolarized *V*_rest_ (Fig. 2*B*) rendering lower levels of steady‐state subthreshold inactivation of VGSCs in *Snord116del* neurons. Combined data in Fig. 2 indicate that pyramidal neurons in the ACC of *Snord116del* mice are less sensitive to the excitatory input noise than WT neurons due to more hyperpolarized *V*_rest_, which would reduce the probability of spontaneous AP firing. This factor in combination with generally higher amplitudes of the APs generated by *Snord116del* neurons might result in more efficient signal‐to‐noise discrimination of the excitatory inputs in the ACC of the *Snord116del* compared to WT mice.

### *Snord116del* neurons are deficient in subthreshold depolarizing conductance

**Figure 3:**
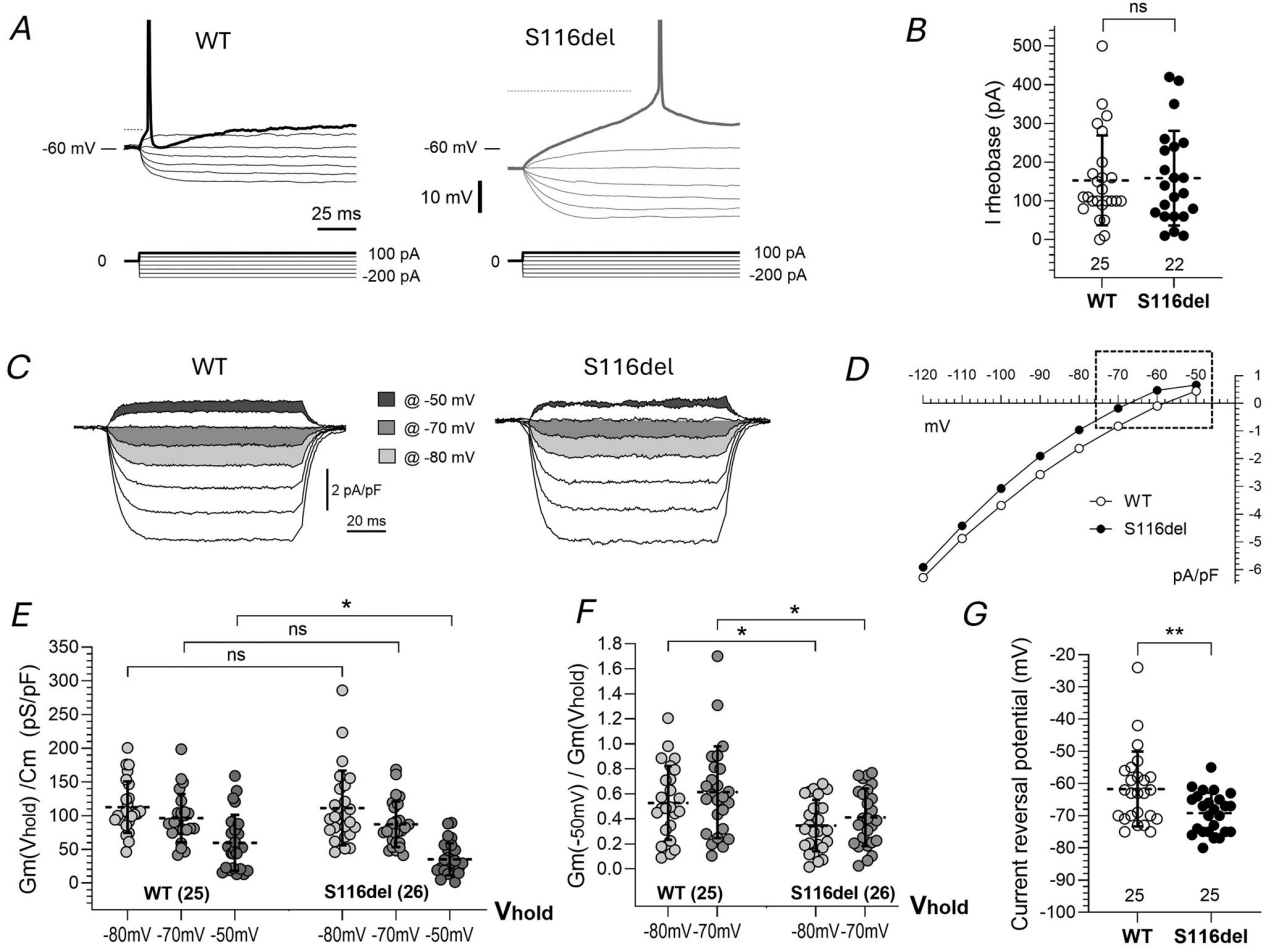
*Snord116del* neurons are deficient in subthreshold depolarizing conductance *A*, incremental current steps used to evoke an action potential (AP) and to calculate rheobase (*I*_rhe_) in representative current‐clamped WT and *Snord116del* neurons. *B*, statistics of *I*_rhe_ in WT and *Snord116del* neurons. *C*, traces of *C*_m_‐normalized membrane currents (*i*) generated in response to 10 mV Δ*V*_m_ steps (−120 through −50 mV) in representative voltage‐clamped WT and *Snord116del* neurons reveal similar chord conductances at *V*_m_ −80 and −70 mV (light grey areas), and deficient conductance in *Snord116del* neuron at *V*_m_ −50 mV (dark grey areas). *D*, i−*V*_m_ characteristics of currents presented in panel *C*. *E*, statistics of *C*_m_‐normalized chord conductance (*G*_m_) in WT and *Snord116del* neurons at *V*_m_ −80, −70 and −50 mV. *F*, statistics of the ratio *G*_m_(−50 mV)/*G*_m_(*V*_hold_) for two *V*_hold_ levels of −80 and −70 mV calculated for each experimental WT and *Snord116del* neuron. *G*, statistics of the total membrane current reversal potential (*V*_rev_) reveal hyperpolarized shift in *Snord116del* relative to WT neurons. The number of tested cells is indicated at the bottom, mean values are represented by dashed lines on the graphs and error bars represent ± SD. *P < 0.05, **P < 0.01 (see numerical and *P* values in the text).

The larger total membrane surface and lower *R*_in_ in *Snord116del* neurons (Fig 1*B,C*) predict higher rheobase (*I*_rhe_, the lowest steady input current necessary to fire a single AP) for these neurons, as it was reported for other hypertrophic neurons with increased *C*_m_ (Cullen et al., 2023; Nguyen et al., 2024; Tariq et al., 2022). A larger voltage barrier for AP firing (Fig 2*D*) would corroborate the predicted increase in *I*_rhe_ for *Snord116del* neurons. We measured apparent chord *I*_rhe_ values in current‐clamped neurons using an approximation calculation formula (see Methods) upon incremental current injections (Δ*I* = 50pA, 500 ms) from the *V*_rest_ level. Surprisingly, we found that apparent *I*_rhe_ was slightly but not significantly increased in *Snord116del* neurons compared to controls (Fig. 3*B*; WT: 153 ± 116 pA (*n* = 25), *Snord116del*: 159 ± 122 pA (*n* = 22), *P = *0.962). A possible explanation for the apparent lack of difference in *I*_rhe_ between two genotypes might be the absence of somal hypertrophy of *Snord116del* neurons despite the increased total membrane surface. Another possibility might reflect a differential pattern of expression of certain subthreshold voltage‐dependent membrane conductances in *Snord116del* neurons that compensates for lower *R*_in_ at rest and for generally higher voltage barrier (*V*_thr_–*V*_rest_) to fire the first AP.

To explore the pattern of subthreshold conductances in both genotypes, we first measured (in the voltage‐clamp mode) the membrane current density (*i = I*_m_/*C*_m_) at various *V*_m_ within −120 to −50 mV range (Fig. 3*C*). We found that at *V*_m_ held at −80 and −70 mV (∼*V*_rest_ range), the normalized membrane chord conductance (*G*_m_/*C*_m_) was not significantly different between WT and *Snord116del* neurons (Fig. 3*E*), ((−80 mV) WT: 113 ± 38 pS/pF (*n* = 25), *Snord116del*: 111 ± 55 pS/pF (*n* = 26), *P = *0.908; (−70 mV) WT: 96 ± 36 pS/pF (*n* = 25), *Snord116del*: 87 ± 34 pS/pF (*n* = 26), *P = *0.410). However, *G*_m_/*C*_m_ was significantly smaller for *Snord116del* than for WT neurons at *V*_m_ = −50 mV (Fig. 3*E*; WT: 60 ± 42 pS/pF (*n* = 25), *Snord116del*: 35 ± 24 pS/pF (*n* = 26) *P = *0.0280). This resulted in a visible flattening of the current‐voltage curve (i−V) for *Snord116del* neurons at *V*_m_ approaching ∼*V*_thr_ range, whereas the same curve for WT neurons remained quasi‐linear (boxed area in Fig 3*D*). The ratios of *G*_m_(−50 mV)/*G*_m_(−80 mV) and *G*_m_(−50 mV)/*G*_m_(−70 mV) calculated separately for each individual neuron showed a genotype‐specific significant decline in *Snord116del* compared to WT neurons (Fig. 3*F*), ((*V*_hold_ −80 mV) WT: 0.527 ± 0.295 pS/pF (*n* = 25), *Snord116del*: 0.346 ± 0.205 pS/pF (*n* = 26), *P = *0.0230; (*V*_hold_ −70 mV) WT: 0.614 ± 0.366 pS/pF (*n* = 25), *Snord116del*: 0.412 ± 0.230 pS/pF (*n* = 26), *P = *0.0117). The results in Fig. 3*C–F* indicate that subthreshold K^+^ conductances (typically the inward‐rectifying Kir and leak K2P) contributing to *V*_rest_ are similarly active in both genotypes at *V*_rest_ more negative to −60 mV, but an additional conductance, missing in *Snord116del*, is active only in WT neurons at *V*_rest_ more positive to −60 mV. To gain insight into the nature of the additional *G*_m_ component in WT neurons, we measured the reversal potential for the *i*−V curves (*V*_rev_) in individual neurons and found that *V*_rev_ values are right‐shifted for WT relative to *Snord116del* neurons (Fig. 3G; WT: −61.7 ± 11.6 mV (*n* = 25), *Snord116del*: −69.2 ± 6.3 mV (*n* = 25), *P = *0.0087), indicating that a persistent depolarizing conductance is normally present in WT but is missing in *Snord116del* neurons at the *V*_m_ range close to *V*_thr_. On one hand this explains more negative *V*_rest_ values obtained in *Snord116del* than in WT neurons (Fig. 2*B*). On the other hand a missing subthreshold conductance in *Snord116del* neurons increases the *R*_in_ and provides a steeper voltage rise (d*V*_m_/d*I*_m_) while approaching *V*_thr_. This property effectively equilibrates the *I*_rhe_ values for both genotypes (Fig. 3*B*) despite a more negative *V*_rest_ in *Snord116del* neurons, providing similar efficiency in firing an AP (i.e. similar intrinsic neuronal soma excitability) in response to a sustained excitatory input.

### *Snord116del* neurons have stronger low‐pass filtering of the excitatory input

**Figure 4:**
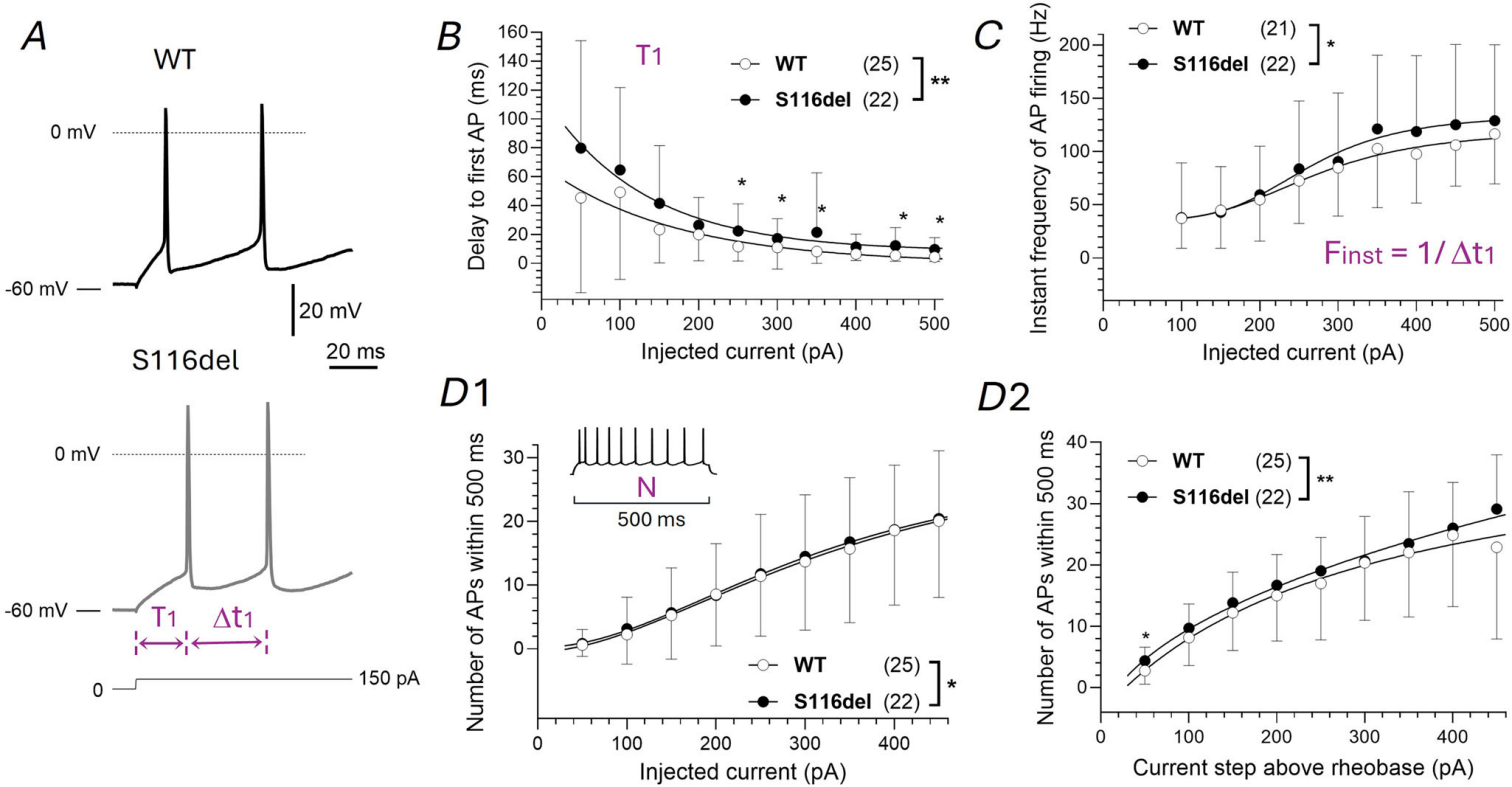
*Snord116del* neurons have stronger low‐pass filtering of the excitatory input *A*, traces of the initial segment of the action potential (AP) trains evoked by low‐amplitude suprathreshold current injection (150 pA) in representative current‐clamped WT and *Snord116del* neurons that highlight the *T*_1_ and Δ*t*_1_ parameters. *B*, the delay before firing the first AP (*T*_1_) is significantly longer for *Snord116del* than for WT neurons over the entire range of injected current steps. *C*, the instant frequency of AP firing derived from the first interspike interval (1/Δ*t*_1_) is significantly higher for *Snord116del* than for WT neurons over the entire range of injected current steps. *D1*, the overall ‘runaway’ frequencies of firing within a train of APs evoked by 500 ms‐long current injection steps (I_inj_) up to 450 pA are slightly higher in *Snord116del* neurons, but (*D2*) same graphs plotted against current injection steps above the rheobase level (Δ*I* = I_inj_ − *I*_rhe_) are visibly and significantly higher for *Snord116del* than for WT neurons. Statistical significance markers (*P < 0.05, **P < 0.01) above traces correspond to the individual points within a set (Mann‐Whitney test); markers next to the genotype identifiers represent the number of experiments and results of the Wilcoxon test comparison between the entire sets (*Snord116del vs*. WT) (see numerical and *P* values in the text).

Cortical pyramidal neurons *in vivo* receive tonic excitatory stimulation at rest at about the rheobase level, resulting in periodic spontaneous firing of single APs. Synchronous phasic activation of multiple inputs provides higher excitatory ionic current resulting in somatic discharge of AP bursts, the pattern of which defines a mode of plasticity in efferent and afferent synapses. We examined whether WT and *Snord116del* pyramidal neurons in ACC generate different patterns of AP trains in response to depolarizing current injections. First we analysed the delay to first AP (*T*_1_) and instant firing frequency (*F*_inst_, the inverted first interspike interval 1/Δ*t*_1_) depicted in Fig. 4*A*. Longer *T*_1_ manifests the ability for the neuron to filter out short bouts of incoming excitation and to fire bursts of APs only in response to longer and stronger phasic bouts. We found that *T*_1_ values were persistently and significantly longer in *Snord116del* neurons (Fig. 4*B*; WT: 18.5 ± 16.4 ms (*n* = 25), *Snord116del*: 30.7 ± 24.0 ms (*n* = 22), Wilcoxon test: *P = *0.0020) while the Δ*t*_1_ intervals were shorter, resulting in significantly higher *F*_inst_ values in *Snord116del vs*. WT neurons (Fig. 4*C*; WT: 79.8 ± 28.8 Hz (*n* = 21), *Snord116del*: 90.0 ± 36.2 Hz (*n* = 22), Wilcoxon test: *P = *0.0117). These results indicate that ACC pyramidal neurons in *Snord116del* mice would be more resistant to fire bursts of APs in response to brief (< 20 ms) bouts of input activity, but if fired, the bursts might be of higher frequency in *Snord116del* compared to WT neurons.

We also measured a conventionally reported maximal (runaway) AP firing frequency by counting the number of APs generated in response to long (500 ms) suprathreshold current injections up to 500 pA. For both genotypes, the graphs of runaway AP frequency revealed an almost overlapping quasi‐sigmoidal, non‐saturated shape with smallest increase in *Snord116* neurons (Fig. 4*D1*; WT: 10.7 ± 7.0 Hz (*n* = 25), *Snord116del*: 11.1±7.0 Hz (*n* = 22), Wilcoxon test: *P = *0.0117). However, when the number of APs was averaged only for current steps above the rheobase level, the *Snord116del* neurons fired APs persistently at a significantly higher rate (Fig. 4D2; WT: 16.2±7.4 Hz (*n* = 25), *Snord116del*: 18.1 ± 7.9 Hz (*n* = 22), Wilcoxon test: *P = *0.0039) indicating that the intrinsic regenerative mechanism for AP firing may be faster in *Snord116del* neurons.

### *Snord116del* neurons have firing patterns favourable for stronger synaptic release

**Figure 5:**
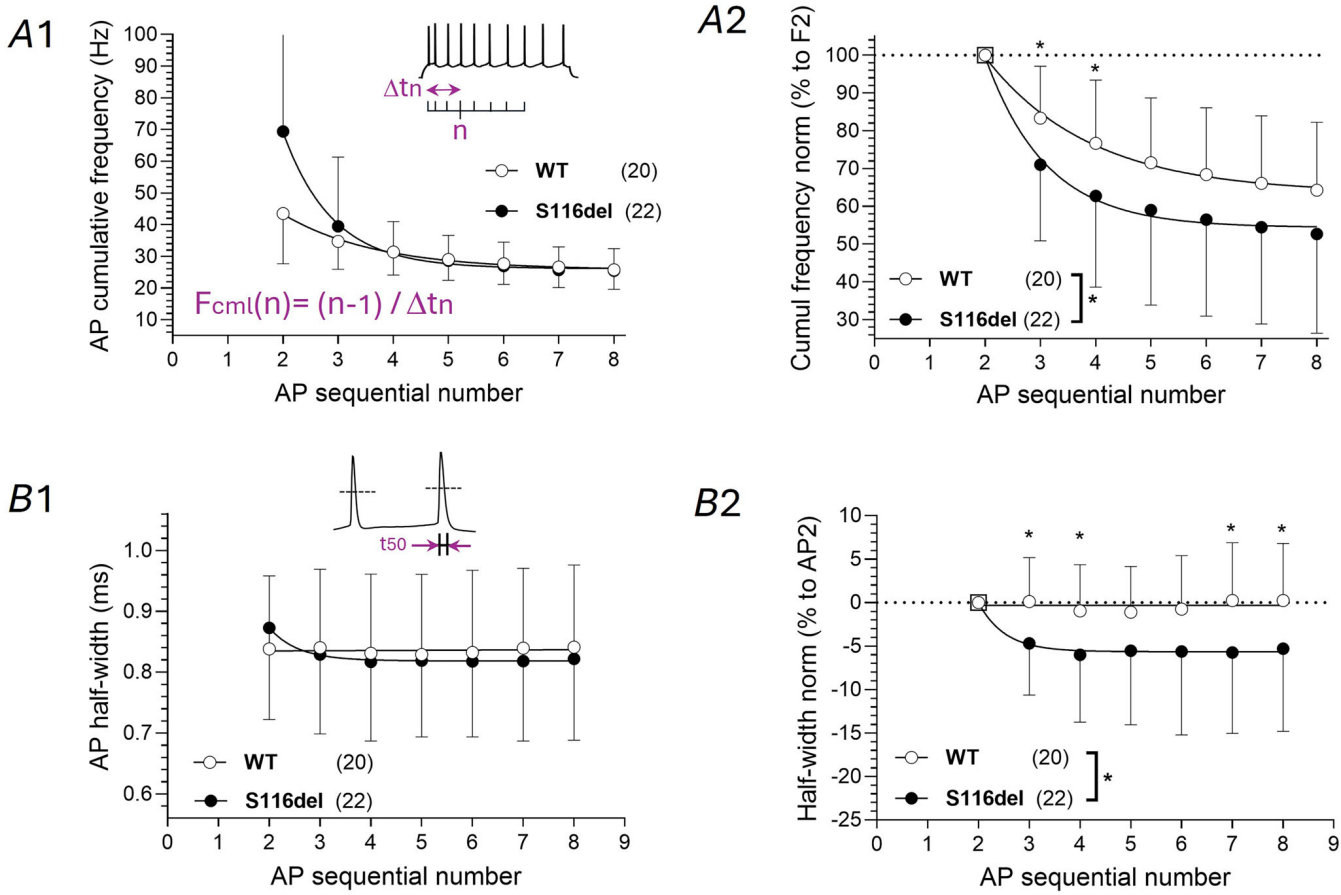
*Snord116del* neurons have firing patterns favourable for stronger synaptic release *A1*, analysis of cumulative frequency (*F*_cml_(*n*), see Methods and insert at the top) of action potential (AP) firing within trains composed of eight APs generated by 500 ms low‐suprathreshold current injection pulses in current‐clamped WT and *Snord116del* neurons. *A2*, *F*_cml_(*n*) data from panel A1 normalized by the initial values of *F*_cml_(2) for both genotypes. *B1*, analysis of the AP half‐width (*t*_50_(*n*)) for each consecutive AP within a train of eight, obtained in WT and *Snord116del* neurons. *B2*, *t*_50_(*n*) data from panel B1 normalized by the initial values of *t*_50_(2) for both genotypes. Statistical significance markers (*P < 0.05) above traces correspond to the individual points within a set (Mann‐Whitney test); markers next to the genotype identifiers represent the number of experiments and results of the Wilcoxon test comparison between the entire sets (*Snord116del vs*. WT) (see numerical and *P* values in the text).

Maximal theoretical runaway AP firing rates generated by neurons upon overloading current injections (as applied above in Fig. 4 *D1*/*D2*) are not attainable in physiological conditions due to much more subtle levels of tonic or phasic synaptic excitatory inputs *in vivo* compared to standard experimental protocols assessing general membrane excitability. Instead in response to mild physiological depolarizing current injections, cortical neurons demonstrate spike frequency adaptation, a gradual reduction in AP frequency within a train. We examined the AP firing adaptation patterns of ACC neurons from WT and *Snord116del* mice by counting parameters of eight initial APs appearing on the first trace showing at least eight APs in response to the lowest 500 ms current step above the *I*_rhe_ level. First we calculated the running cumulative frequency as a function of the number of APs within a burst (*F*_cml_(*n*), defined in the Methods section and illustrated in Fig. 5*A1*) with *n* running from 2 to 8. Predictably *F*_cml_(2) (similar to *F*_inst_ in Fig. 4*C*), was markedly higher for *Snord116del* neurons, corroborating our results in Fig. 4*C*. With increasing *n* the *F*_cml_(*n*) shows a progressive decline due to spike frequency adaptation, but at different rates for two genotypes. The sharply declining *F*_cml_ curve for *Snord116del* intersects moderately declining WT *F*_cml_ curve at *n = *4 (Fig. 5*A1*; WT: 31.2 ± 6.2 Hz (*n* = 20), *Snord116del*: 35.3 ± 15.8 Hz (*n* = 22), Wilcoxon test: *P* > 0.999). To emphasize the differences in frequency adaptation in two genotypes, we normalized both sets by their initial *F*_cml_(2) values, which revealed a significantly faster *F*_cml_ decline within a train of APs in neurons from *Snord116del* compared to WT mice (Fig. 5*A2*; WT: 75.8 ± 12.5 % (*n* = 20), *Snord116del*: 65.2 ± 15.5 % (*n* = 22), Wilcoxon test: *P = *0.0312).

Next, we analysed changes in the AP width within a sequence of eight APs, measured at half‐amplitude for each AP (*t*_50_) for both genotypes. In WT neurons the *t*_50_ was relatively stable during the entire train. In *Snord116del* neurons the initially larger APs narrowed abruptly after *n = *2, with *t*_50_ stabilizing at a slightly lower level compared to WT neurons (Fig. 5*B1*; WT: 0.836 ± 0.005 ms (*n* = 20), *Snord116del*: 0.828 ± 0.020 ms (*n* = 22), Wilcoxon test: *P = *0.297). Normalizing the *t*_50_ values by the initial values *t*_50_(2) for both genotypes revealed nearly absent AP width adaptation in WT, but a quick and significant reduction in the AP width in *Snord116del* neurons (Fig. 5*B2*; WT: −0.31 ± 0.60 % (*n* = 20), *Snord116del*: −4.69 ± 2.11 % (*n* = 22), Wilcoxon test: *P = *0.0312). The data presented in Fig. 5 indicate that ACC neurons in *Snord116del* mice are capable of firing at higher rates than in WT neurons during short bursts composed of 3–4 APs. This ability combined with the larger width of the initial AP favours conditions for stronger synaptic release and synaptic potentiation in axon terminals of *Snord116del* compared to WT neurons produced by short bursts of APs generated by neuronal soma.

### Membrane resting state determines AP spiking patterns in WT and *Snord116del* neurons

**Figure 6:**
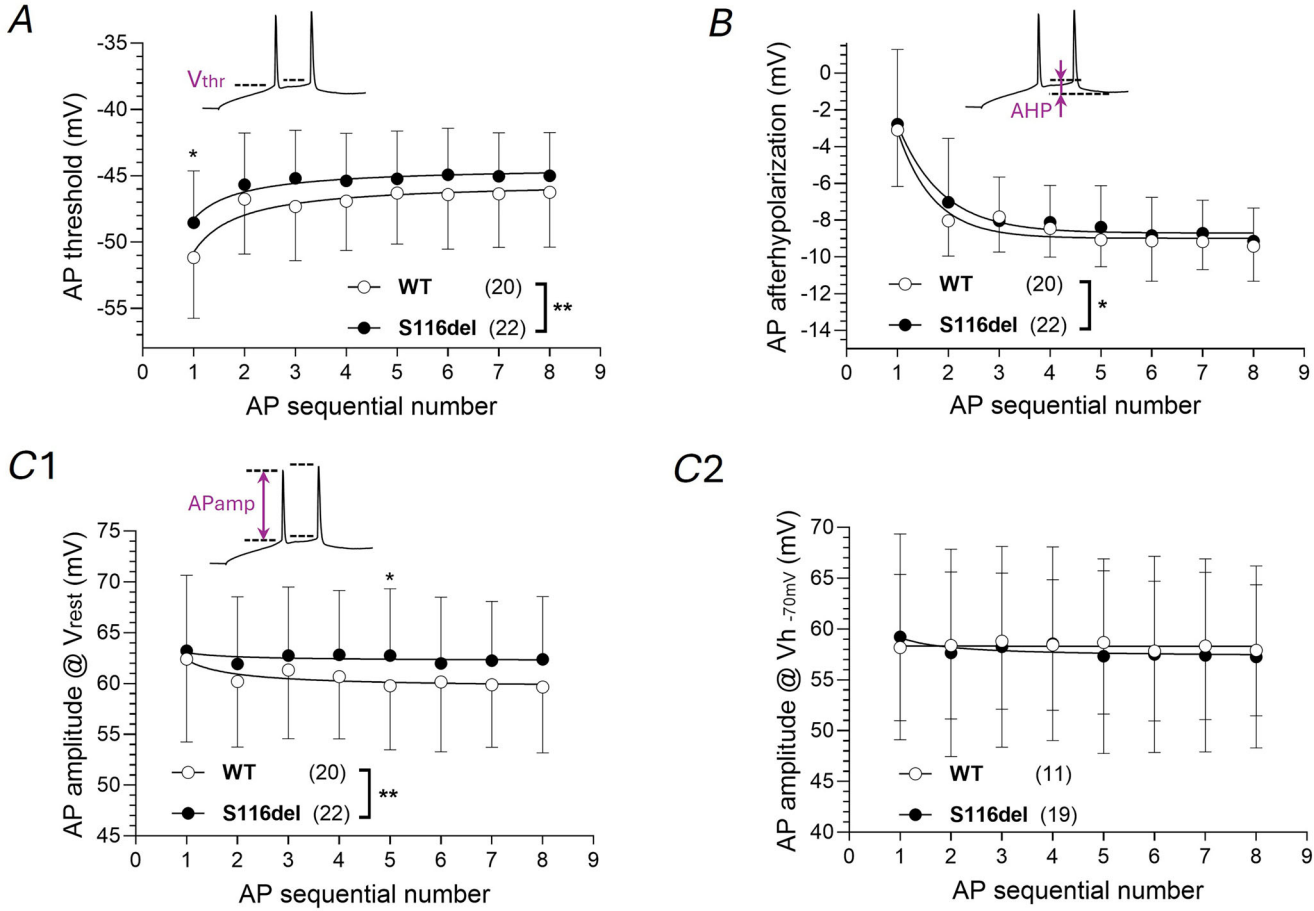
Membrane resting state determines action potential (AP) spiking patterns in WT and *Snord116del* neurons *A*, higher extent of induced inactivation of voltage‐gated sodium channels (VGSCs) (see main text) determines significantly more depolarized *V*_thr_ for action potentials (APs) generated by low‐suprathreshold current in *Snord116del* neurons compared to WT. *B*, afterhyperpolarizations following regenerative APs are reduced in *Snord116del* neurons compared to WT. *C1*, amplitudes of regenerative APs in *Snord116del* neurons are stable and larger than amplitudes of WT neuron APs, which experience early rundown. *C2*, holding neuronal resting membrane potential for both genotypes at the same intermediary level *V*_h_ = −70 mV eliminated AP_amp_ differences between genotypes. Statistical significance markers (*P < 0.05, **P < 0.01) above traces correspond to the individual points within a set (Mann‐Whitney test); markers next to the genotype identifiers represent the number of experiments and results of the Wilcoxon test comparison between the entire sets (*Snord116del vs*. WT) (see numerical and *P* values in the text).

Using moderate physiological‐level current pulses helped to reveal differences in *T*_1_ between two genotypes (Fig. 4*A,B*) that were masked in the early experiments applying strong suprathreshold current pulses (Fig. 2*A*). Smaller injected currents generate longer ascending membrane potential ramps in the soma, which induce partial subthreshold inactivation of the VGSCs before the first AP is fired. Biophysical studies revealed that higher levels of subthreshold inactivation result in increased *V*_thr_ for VGSC activation and in reduced amplitude of subsequently generated inward sodium currents (Carr et al., 2003; Groome et al., 2011; Venkatesan et al., 2014). We suggested that longer *T*_1_ in *Snord116del* neurons would result in a higher level of subthreshold inactivation, and consequently in a higher *V*_thr_ for AP1 compared to WT neurons. We applied the same experimental protocol as in Fig. 5 and found that (contrary to results in Fig. 2C) the *V*_thr_ value for AP1 was indeed higher in *Snord116del vs*. WT neurons at physiological levels of injected current, and this significant difference persisted for all eight APs in the train (Fig 6A; WT: −47.2 ± 1.7 mV (*n* = 20), *Snord116del*: −45.6 ± 1.2 mV (*n* = 22), Wilcoxon test: *P = *0.0078). Further analysis revealed that the persistence of the elevated *V*_thr_ values within a train of APs might be explained by a little, but statistically a significant deficit in generating the afterhyperpolarizations (AHP) within a train of APs in *Snord116del* neurons (Fig. 6*B*; WT: −8.0 ± 2.1 mV (*n* = 20), *Snord116del*: −7.6 ± 2.1 mV (*n* = 22), Wilcoxon test: *P = *0.0156) leading presumably to a higher level of slowly developing steady‐state inactivation of VGSCs in *Snord116del* compared to WT neurons during a sustained depolarizing input.

The higher level of VGSC subthreshold inactivation may also reduce the amplitude of the initial AP. We examined the pattern of AP_amp_ in the datasets from Fig. 6*A,B*. Contrary to the results in Fig. 2*E*, the amplitude of the AP1 in *Snord116del* neurons was indeed reduced to the level close to that in WT neurons. However the AP_amp_ dynamics within a train were different between two genotypes. The AP amplitudes remained stable for the entire train in *Snord116del* neurons, whereas a progressive decrease in AP_amp_ was observed in WT neurons, making the overall AP_amp_ values within a group significantly lower for WT than for *Snord116del* neurons (Fig. 6*C1*; WT: −60.5 ± 09 mV (*n* = 20), *Snord116del*: −62.5 ± 0.5 mV (*n* = 22), Wilcoxon test: *P = *0.0078). To examine whether different dynamics of AP_amp_ within a train may result from the inherently different *V*_rest_ between WT and *Snord116del* neurons (Fig. 2*B*), we repeated the last series of experiments in conditions where the naturally occurring *V*_rest_ values for two genotypes were mechanistically equalized. In the current‐clamp mode of recording, we injected additional current at rest to adjust and hold the membrane potential at the same (intermediary with respect to *V*_rest_) level of *V*_hold_  = −70 mV for all experimental neurons. This artificial manipulation brought the AP_amp_ curves for two genotypes towards each other and eliminated the significant difference between two sets (Fig. 6*C2*; WT: −58.3 ± 0.3 mV (*n* = 11), *Snord116del*: −57.9 ± 0.7 mV (*n* = 19), Wilcoxon test: *P = *0.195). Besides the shapes of the AP_amp_ curves for two genotypes flipped: the WT curve flattened, whereas the *Snord116del* curve revealed a mild tendency for rundown. This experiment revealed that differentially expressed subthreshold ion conductances that separate WT and *Snord116del* neurons in terms of their *V*_rest_ (more negative in *Snord116del*) extend their causal influence to differentially modulate transient ion currents that shape regenerative APs in WT and *Snord116* neurons during AP bursts. The resulting differential spiking patterns of AP bursts may determine different rules for neural network plasticity within the ACC for two genotypes, leading to specific behaviour traits controlled by this brain area in WT and *Snord116del* mice, including feeding behaviour.

## Discussion

### *Snord116* deficiency may optimize food‐seeking strategies in mice

Our work revealed that multiple biophysical properties and predicted morphological features of the layer 2/3 pyramidal neurons in the ACC brain area differ in WT and *Snord116del* mice sufficiently to expect distinct behavioural outcomes between two genotypes. Paradoxically, some of our findings in *Snord116del* ACC, namely a higher density of excitatory inputs, predictably better signal‐to‐noise discrimination, a higher low‐pass filtering of the excitatory inputs by the soma and higher frequency for short AP burst discharges, may be associated with higher neuronal network processing efficacy in the ACC of the *Snord116del* compared with WT mice. In this regard our data may support previous reports on the absence of obvious defects in working or spatial memory in *Snord116del* mice (Ding et al., 2008; Scott et al., 2025; Zieba et al., 2015). In rodents as in humans the ACC encodes preparatory attention and error detection. Lesions in this area significantly decrease accuracy during attention tasks in rats and enhance probability of premature responses (Wu et al., 2017). The augmented neural network processing in the ACC of the *Snord116del* mice predicted by our work may provide a currently missing neuronal basis for the unexplained paradoxical observation that *Snord116del* mice perform significantly better than WT in the working‐for‐food task by producing lower error rate of timed behaviours, which allow them to develop an optimal strategy to gather more food resulting from better decision making (Lassi, Maggi et al., 2016).

Interestingly, another PWS mouse model carrying the deletion of *Ndn* gene proximal to *Snord116* cluster produced a noradrenergic neuron phenotype in the locus coeruleus characterized by lower spontaneous firing rates and delayed AP firing upon depolarizing current injections (Wu et al., 2020) that is similar to our observations for *Snord116del* ACC pyramidal neurons. There exists a possibility that the normal transcription of *Ndn* gene is modulated by molecular interactions with regulatory Snord116 RNA within a proximal ‘grape‐like’ Snord116 RNA cloud tethered to the site of *Snord116* transcription (Vitali et al., 2010), which co‐purifies with transcriptional activator RBBP5 (Powell et al., 2013). In this scenario Snord116 RNA can influence neuronal properties either directly by controlling transcription of targeted genes or indirectly by modulating the *Ndn* RNA expression level. Notably, the whole brain *Ndn* RNA expression significantly decreased in neonatal whole brain of *Snord116del* mice detected via RNAseq tests (Zahova et al., 2021, Table S3). Corroborating a hypothesis about a possible *Snord116*/*Ndn* transcriptional coordination was a report that similar to *Snord116del* mice overperforming the WT during the working‐for‐food task (Lassi, Maggi et al., 2016), the *Ndn* deficient mice demonstrated an improved spatial learning and memory in the Morris water maze compared to WT mice (Muscatelli et al., 2000).

### PWS hyperphagia may be linked to *Snord116/SNORD116* deficiency in the ACC

Our data about possible altered neural network processing efficacy in the ACC of the *Snord116del* mice implies that similar neuronal abnormalities may take place in matching human brain areas of the *SNORD116*‐deficient PWS patients. In humans the ACC is involved in decision making, including, among others, control of feeding behaviour. One can speculate that abnormally high excitatory activity or enhanced network processing efficacy in this area may contribute to symptomatic hyperphagia in PWS. There exist at least three lines of evidence to support this hypothesis. *First, the ACC is a major cortical area that processes hedonic value of food items*. The early studies measuring regional cerebral blood flow (rCBF) discovered that ACC is one of the few cortical brain areas whose activity is increased during a state of hunger (Tataranni et al., 1999). Later studies using fMRI revealed that cingulate gyrus and insula were the only areas that met individual statistical criteria of significant activation upon visual and tactile presentation of food items, linking these areas to conscious hedonic perception of food value (St‐Onge et al., 2005). Similar studies revealed that a significant interaction between the states of hunger and satiety and stimulation with food and nonfood images occurred in the ACC, superior occipital sulcus and the right amygdala (Fuhrer et al., 2008). It was suggested that the essential link between homeostatic state and hedonic drive to eat is conveyed by polysynaptic efferent connections from chemosensory neurons in the area postrema to a cingulate cortex and insula (McKinley et al., 2019). *Second, the ACC is the executive hub of the reward system that governs a context‐induced compulsive seeking of addictive attractants, including food*. The orbitofrontal cortex and ACC in humans encode the cognitive reward value of taste that modulates and overrides existing satiety signals, and human obesity is associated with overstimulation of this reward network (Rolls, 2005, 2012). Dorsal ACC is hyperactive in patients with OCD (McGovern & Sheth, 2017), and one of the main debilitating features in PWS is compulsive eating which results from impaired cognitive control. Similarly, a specific ACC neuronal subgroup was found to be involved in the addictive drive in mice, particularly the layer 5 ACC neurons projecting to the VTA area. These neurons became more active during the development of the context‐induced substance‐seeking behaviour that was significantly reduced by their targeted inhibition (McKendrick et al., 2022). *Third, the neuroimaging studies revealed altered neural function in multiple cortical areas of PWS subjects (* *Manning & Holland*, 2015 *), with ACC predominantly showing signs of augmented activity*. The early PET imaging studies in PWS patients showed contradictory results reporting a reduced rCBF in the ACC in one study (Mantoulan et al., 2011), but an increased glucose metabolism in the ACC and multiple frontal cortex areas in another (Kim et al., 2006). Interestingly, the results of both studies can be supported by our data on various aspects of functional state of mouse ACC *Snord116del* neurons, predicting lower neuronal energy expenditure during inactive state (lower spontaneous firing due to more hyperpolarized *V*_rest_), but higher energy expenditure during task‐related ACC activation (stronger synaptic excitatory input and higher intrinsic frequency of APs within bursts found in our study). Accordingly, the latter study (Kim et al., 2006) showing an increased ACC metabolic rate in PWS patients relative to controls was conducted on subjects fasting for more than 4 h leading to a higher probability for food‐seeking internal brain activity. More recent fMRI studies revealed an increased overall activation and reduced deactivation upon task switching in the anterior ventromedial PFC (includes OFC and ACC) in PWS subjects compared to control group (Woodcock et al., 2010). Similar fMRI studies revealed increased activity in the PFC, ACC, parietal lobe and insula in PWS patients compared to the control group (Zhang et al., 2013). Following study from the same group revealed significantly enhanced functional connectivity from MPF and ACC to the amygdala, and from the amygdala to the hypothalamus in the PWS group that suggests the existence of a strong driving force from the higher cortical regions via amygdala to the hypothalamus that might potentially override metabolic signals of satiety in food‐related circuitry and lead to extreme hyperphagia in PWS patients (Zhang et al., 2015). Combining our results on specific changes in biophysical properties and increased excitatory synaptic connectivity of L2/3 neurons in the mouse ACC brain area of *Snord116del* mice with literature data reviewed above favours a novel hypothesis on the existence of a significant hedonic component in hyperphagia in PWS patients driven by enhanced involvement of the ACC and probably other components of the food reward system, provided that neuronal abnormalities found in our study on *Snord116del* mice occur in human brain cortical arears affected by *SNORD116* deficiency.

### Expression of mTOR pathway cortical genes is altered by *Snord116* deficiency

The significant functional and predicted morphological aberrations in *Snord116del* mouse neuronal parameters found through our study may originate from missing nucleolar *Snord116* RNA ‘cloud’ structures found around the point of transcription of multiple target genes, thereby disrupting their normal expression. The Snord116 RNA clouds are found in multiple locations within neuronal nuclei in primary neuron cultures (Vitali et al., 2010) and in WT adult mice (Powell et al., 2013). We analysed a series of RNAseq databases obtained previously in PWS subjects and in *Snord116del* mice, to gain insight into the sets of protein‐encoding genes that may be altered by *Snord116* deficiency (Table S3 Genomic Databases).

Our findings of increased membrane surface (higher *C*_m_) and excitatory synaptic density (higher sEPSC frequency) in *Snord116del* neurons resemble a well‐documented neuronal hypertrophy phenotype caused by overactive intracellular mTOR signalling pathways. Our electrophysiological data corroborate the earlier report about cortex‐specific increased mTOR activity in *Snord116del* mice not observed in the hippocampus (Powell et al., 2013). Interestingly, our extensive search within previously published RNAseq databases revealed that contrary to the cortical tissue, expression profiles for multiple members of mTOR signalling cascade were normal in the hypothalamic tissue samples collected both from human PWS patients (Bochukova et al., 2018) and from conditional (Polex‐Wolf et al., 2018) and congenital (Knott et al., 2022) *Snord116del* mice. Two branching mTOR signalling pathways, mTORC1 and mTORC2 differentially influence morphology of perisomatic and dendritic neuronal compartments, with mTORC2 preferentially shaping distal dendritic regions (Chen et al., 2019; Cullen et al., 2023). Based on our data we speculate that mTORC2 pathway, rather than mTORC1, may be enhanced by *Snord116* deficiency. *First*, in accordance with previously reported smaller forebrain neuronal soma sizes found previously in *Snord116del* mice (Burnett, Hubner et al., 2017), we believe that somatic compartment may indeed not be enlarged in *Snord116del* ACC neurons, as the rheobase values were not markedly increased (Fig. 3*B*) and AP_amp_ was higher (Fig 2*E*) in *Snord116del* ACC neurons in our experiments. The opposite effect of increased rheobase and decreased AP_amp_ was described recently in mTORC1‐overactive cortical neurons characterized by enlarged soma (Nguyen et al., 2024). *Second*, the increased sEPSC frequency in *Snord116del* neurons found in our work resembles the effect of *Pten* deficiency in mouse forebrain‐ and dentate gyrus neurons, the effect originating from the overgrown dendritic tree (Tariq et al., 2022). This effect of increased sEPSC frequency was rescued by mTORC2 but not mTORC1 inhibition (Chen et al., 2019). Our analysis of differentially expressed genes (DEGs) in mouse *Snord116del* cortex from Powell et al. RNAseq database revealed that, depending on time of the day, both activators (Rheb, Rptor, Akt3) and repressors (Pten, Depdc5, Tsc2, Deptor) of the mTORC1 pathway are simultaneously upregulated and potentially balance each other in *Snord116del* neurons, whereas the expression of the mTORC2 pathway activator Rictor was unopposed (Powell et al., 2013). Thus, currently available genomic databases support our hypothesis that ACC pyramidal neurons in *Snord116del* mice might have an overgrown dendritic tree, but a normal or even smaller soma due to an unbalanced activation of mTORC2 pathway. Future morphological studies and molecular biology tests on the status of mTOR signalling pathways in cortical neurons of *Snord116del* mice are necessary to verify our assumptions.

### Increased expression of multiple ion channel genes in *Snord116del* alters cortical neuron properties

To make sense of other molecular aberrations resulting in *Snord116del* neuronal electrophysiological phenotype revealed through our study, we analysed differentially expressed genes encoding membrane ion channels in eight transcriptome databases originating from previously published clinical PWS human‐ and *Snord116del* mouse model studies (Table S3). Interestingly, the altered gene expression patterns in PWS hypothalamic samples (both human and mice) (Bochukova et al., 2018; Knott et al., 2022; Polex‐Wolf et al., 2018) and mouse embryonic whole brain samples (Zahova et al., 2021) differed from patterns obtained in adult mouse cortical samples (Powell et al., 2013). The latter, in turn, had a partial overlap with altered genes in human PWS adipose‐derived mesenchymal stem cells (hAdMSCs) (Chao et al., 2022) capable of pharmacologically induced transformation into neuronal cells.

About 94% of DEGs from *Snord116del* adult mouse cortex in Powell et al. were upregulated to various extents depending on the Circadian time, but we considered for our analysis only those genes that were significantly upregulated throughout the day. The most relevant group of identified significantly upregulated genes were K^+^ inward rectifier ion channels that may set up a more negative *V*_rest_ in *Snord116del* neurons found through our work: Kir2.1, Kir3.3 and Kir5.1. Notably, Kir2.1 and Kir2.2 are also significantly upregulated in PWS hAdMSCs (Chao et al., 2022). Another upregulated group identified in both *Snord116del* cortical and PWS hAdMSCs databases included genes for K2P K^+^ channels TREK1 and GIRK/KATP‐activating adenosine receptor A1R, both contributing to more negative *V*_rest_ (Gonzalez et al., 2012; Spanoghe et al., 2020).

In our study we found that WT pyramidal neurons possess at rest a subthreshold depolarizing conductance absent in *Snord116del* neurons (Fig. 3*D*). We did not observe functional signature of hyperpolarization‐activated channels (HCN) (Biel et al., 2009) at subthreshold *V*_m_ in WT nor in *Snord116del* neurons (absent characteristic transitory sag upon hyperpolarizing current steps). *HCN* were also absent among DEGs within analysed databases. The expression of genes encoding subthreshold depolarizing low‐threshold activated Ca^2+^ channels (T‐type, CaV3.2, CaV3.3) in PWS/*Snord116del* samples from both mouse cortex and hAdMSCs was upregulated in analysed RNAseq databases, lacking however the expression of functional proteins in neuronal soma based on the opposite, more hyperpolarized *V*_rest_ for *Snord116del* neurons found in our study. Nonetheless, the possibility of higher expression of T‐type Ca^2+^ channels in distal dendrite excitatory synapses (Leresche & Lambert, 2017) that might facilitate synaptic plasticity in *Snord116del* neurons may be addressed in prospective future studies.

The other plausible candidate to carry a subthreshold depolarizing current in WT, but missing in *Snord116del* neurons, would be persistent sodium current with typical activation midpoint around −55 mV (Bean, 2007). In normal conditions, multiple sites at VGSCs are phosphorylated by PKA, PKC and other kinases supporting a measurable persistent sodium current due to a negative shift in voltage dependence of steady‐state inactivation (*V*_h_) and activation (*V*_a_). This effect is opposed by phosphatase 2A and calcineurin (Scheuer, 2011; Scheuer & Catterall, 2006). Our DEG database analysis revealed that phosphatase 2A regulatory β‐subunit, the calcineurin catalytic α‐ and β‐subunits and PKA inhibitory subunit (*Prkar1a*) genes are significantly upregulated in *Snord116del* mouse cortical transcriptome (Powell et al., 2013), as well as Na^+^ channel subunits β (*Scn3b, Scn4b*), which all could shift *V*_h_ and *V*_a_ parameters to more positive membrane potentials (Cummins et al., 2001). Hyperactivity of any (or combined) regulatory mechanisms controlled by these genes in *Snord116del* neurons would result in the suppression of persistent sodium current due to a positive shift in *V*_h_ and transition of VGSCs into a stable closed state at subthreshold membrane potentials, producing more negative *V*_rest_ and multiplicity of other effects observed in Fig. 3*C–G*.

An important finding of our study was the propensity of ACC pyramidal neurons in *Snord116del* mice to fire delayed bursts of APs with higher initial spiking frequency and higher spike frequency adaptation compared to WT neurons (Figs. 4*B,C* and 5*A1*). Various combinations of dozens of voltage‐dependent ion channels in a typical neuron can generate APs with wide ranges of shapes and patterns (Bean, 2007), making it difficult to separate individual contributions of particular ion channels. However it is known that blocking a fast‐inactivating K^+^ current (A‐type) shortens a delay to fire the first AP, whereas reducing a delayed rectifier (M‐type) K^+^ current causes neurons to fire at a higher frequency at the onset of spiking (Connor & Stevens, 1971; Upchurch et al., 2022). In the database presented in Powell et al., gene expression for Kv3.4 (A‐type) is significantly upregulated in *Snord116del* mouse cortex, as well as the expression of K^+^ channel b‐subunit (Kcnab1) that transforms endogenous delayed rectifier M‐type K^+^ channels into the A‐type (Gonzalez et al., 2012). The higher expression of proteins encoded by these genes in *Snord116del* ACC pyramidal neurons may cause a delay in firing the first AP and may produce a higher initial frequency of the AP bursts compared to WT neurons, as found in our experiments (Fig. 4*B,C*). Furthermore, the lower availability of M‐type K^+^ channels may result in shallower AP afterhyperpolarizations (Upchurch et al., 2022), as was seen for *Snord116del* neurons in our study (Fig. 6*B*).

The variable patterns of adaptive transformation of regenerative AP frequency, amplitude, width and threshold originate from a dynamic interplay between closed/open/inactivated states of the dominant Na^+^, and multiple K^+^ channels operating on different time scales. The Na^+^ channel transitions to fast and slow inactivation are the most impactful contributors to dynamic adaptation patterns of APs (Carr et al., 2003; Upchurch et al., 2022; Venkatesan et al., 2014). These transitions strongly depend on the interaction of VGSCs with a group of cytoplasmic receptor‐truncated FGFs 11–14 (alternative names FHF 1–4). Based on mouse cortical neuron transcriptome, the gene expression for FGF12 and FGF14 is significantly increased in *Snord116del* compared to WT neurons (Powell et al., 2013). The higher protein level for these FGFs produces a positive shift in VGSC *V*_h_ (Dover et al., 2010; Lou et al., 2005; Rush et al., 2006) resulting in their stable closure at *V*_rest_ that might explain the apparent suppression of a persistent sodium current in *Snord116del* neurons suggested from our data (Fig. 3*C*–*G*). Additionally this may explain the increase in the amplitude of the first AP in *Snord116del* neurons (Fig. 2*E*) due to a higher percentage of non‐inactivated Na^+^ channels at *V*_rest_ (Rush et al., 2006; Wittmack et al., 2004). An increased level of FGF12 accelerates use‐dependent accumulation of VGSC in a slow inactivated state (Dover et al., 2010; Rush et al., 2006) which would explain a higher values of *V*_thr_ within a train of APs in *Snord116del* neurons (Fig. 6*A*). A recent report on opposite, significantly downregulated expression of FHF member *FGF13* was described in neurons derived from *Snord116del* human embryonic stem cells (Gilmore et al., 2024), although it can be argued that *FGF13* deficiency reported in that study was related to, or caused a global disruption of the VGSC function in these ‘neuronal’ cells, which were not electrically excitable.

The other form of AP adaptation, the use‐dependent shortening of AP width that is stronger in *Snord116del* neurons (*t*_50_, Fig. 5*B1*), may originate from the higher activity of Ca^2+^‐dependent large‐conductance K^+^ channels that contribute prominently to the acceleration of the descending phase of the APs (Gonzalez et al., 2012). At least one of these channels (KCNT1) is highly expressed in the cortex of *Snord116del* mouse compared to controls (Powell et al., 2013). In summary, by analysing available transcriptome databases, we identified candidate upregulated genes that may drive more prominent spike frequency and amplitude adaptations in *Snord116del* compared to WT neurons. Interestingly, it was found in hippocampal CA1 neurons that both frequency and amplitude adaptation of AP spikes were more pronounced in dendritic, compared to somatic compartments (Upchurch et al., 2022). This indicates that our findings of stronger AP spike adaptation in *Snord116del* neurons may partially originate from a stronger relative contribution of a more developed dendritic tree in *Snord116del* neurons predicted from our findings of higher *C*_m_ in combination with higher sEPSC frequency and possibly a more active mTORC2 signalling system in *Snord116del* neurons.

### Altered ACC pyramidal neuron phenotype prompts re‐examination of the *Snord116del* mouse model of PWS

Multiple strands of *Snord116del* mice were developed during the last two decades with a hope to deliver a relevant mouse model of PWS (Kummerfeld et al., 2021; Mendiola & LaSalle, 2021; Salles et al., 2021). Certain somatic hypothalamic aberrations (Burnett, LeDuc et al., 2017; Knott et al., 2022; Pace, Falappa et al., 2020; Polex‐Wolf et al., 2018; Qi et al., 2016) and mild behavioural deviations (Adhikari et al., 2019; Ding et al., 2008; Zahova et al., 2021; Zieba et al., 2015) were indeed found in these mouse models. But a major setback in the field was the inability to reproduce human PWS obese phenotype, particularly in *Snord116del* mice, beyond mild forms of hyperphagia observed when mouse body weights were normalized (Ding et al., 2008; Lin et al., 2014; Osborne‐Lawrence et al., 2023; Qi et al., 2016). Among recent unsuccessful attempts to link *Snord116* deficiency to increased mouse homeostatic feeding drive was a failure to induce obesity in human‐adjusted experimental conditions of ambient thermoneutrality (Osborne‐Lawrence et al., 2023; Qi et al., 2017). In the meantime the alternative possibility that PWS obesity can be driven by increased hedonic feeding drive gained little attention both in human and mouse model studies. The unexplained intriguing observations that *Snord116del* mice demonstrate higher acuity, better decision making and ability to develop improved strategies compared to control mice during working‐for‐food tasks (Lassi, Maggi et al., 2016) indicate that reward‐ and food‐seeking brain circuits that drive hedonic feeding behaviour are strongly engaged in *Snord116del* mice. In rodents, the hedonic feeding drive is specifically activated via augmented neurotensin (NTS) release in the VTA. The overexpression of NTS in NAc‐to‐VTA projecting neurons, besides stimulating hedonic eating, resulted in a particular behavioural phenotype characterized by reduced anxiety score in the open field (OF) tests, and by significant deficit in weight gain while on the high fat diet (HFD) compared to controls (Gazit Shimoni et al., 2025). Surprisingly similar results during OF tests were reported in *Snord116del* and some other PWS mouse models (Ding et al., 2008; Relkovic & Isles, 2013; Zieba et al., 2015), and analogous ‘paradoxical’ HFD‐resistant phenotype was consistently obtained in *Snord116del* mice (Ding et al., 2008; Osborne‐Lawrence et al., 2023; Qi et al., 2016). These parallel findings indicate that hedonic feeding brain circuits may be overactive in *Snord116del* mice similar to VTA/NTS‐overactive mice. This is contrary to the previous negation of possible hedonic mechanism of PWS hyperphagia in one animal study using another (imprinting centre deletion PWS*^ICdel^*) mouse model, and based on reported sucrose preference over non‐caloric saccharin, although being questionable due to the lack of preference for saccharin over pure water by PWS*^ICdel^* mice found in the same study (Davies et al., 2015). Corroborating our hypothesis on potentially increased hedonic feeding drive in *Snord116del* mice, the cortical NTS expression level was significantly increased (+60%, nighttime) in *Snord116del* relative to controls in Powell et al. transcriptome database (Powell et al., 2013).

**Figure 7:**
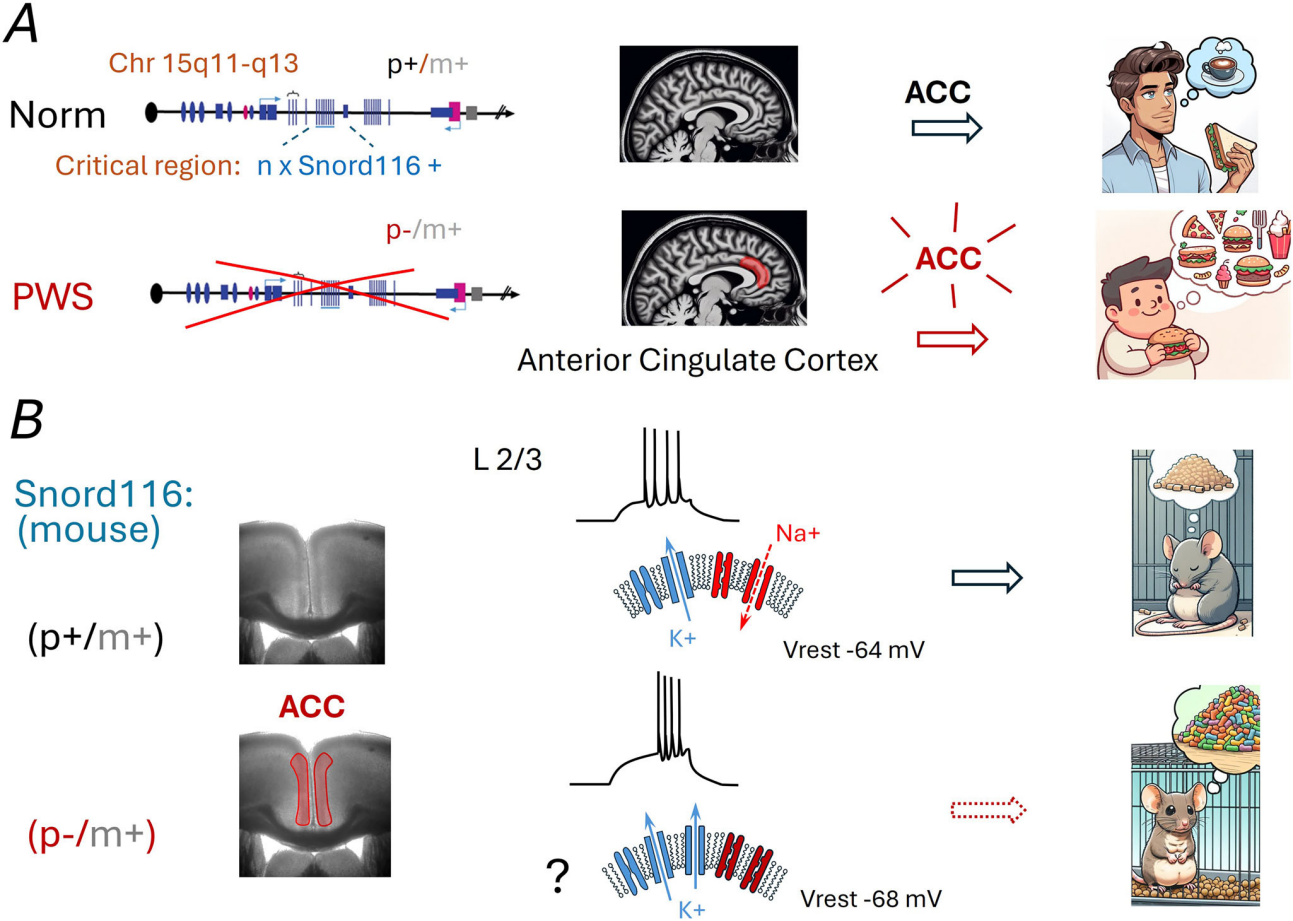
Hedonic hypothesis of the Prader‐Willi syndrome *A*, corrupt critical multiple‐repeat *Snord116*‐containing region of the human paternally imprinted copy on chromosome 15q11‐q13 results among other disturbances, in an overly strong processing in the brain goal‐orienting anterior cingulate cortex (ACC) region leading to an insatiable hedonic‐driven food‐variety seeking behaviour in Prader‐Willi syndrome (PWS) patients, which is distinct from the healthy individuals’ ability to maintain a habitual daily feeding routine. *B*, targeted deletion of the orthologous mouse *Snord116* containing region results in the neuronal phenotype in the ACC L2/3 with better signal‐to‐noise processing power due to lower *V*_rest_ and delayed firing of the high‐frequency short bursts. Presumed neuronal network enhancement in the ACC and in downstream brain areas would strengthen hedonic food‐variety seeking in *Snord116del* mice. In absence of variety, a stronger sensory‐specific satiety (hedonic adaptation) is quickly developed in *Snord116del* mice on highly palatable, but ‘monotonous’ high‐fat chow, which explains a paradoxical high‐fat diet resistant phenotype in *Snord116del* mouse models, resembling feeding behaviour of the hedonic‐overdrive mouse model overexpressing neurotensin (NTS) in the NAc→VTA projecting neurons (Gazit Shimoni et al., 2025).

Feeding behaviour strategizing includes adaptive processing of the action‐outcome prediction and the binary decision making encoded in neural circuits within the ACC and mPFC areas of the brain. Interestingly, the ACC is the cortical area with one of the highest densities of the NTS‐expressing neurons during mouse brain development (Schroeder et al., 2019), when the lifelong feeding preferences are formed. Besides, a specific subgroup of the ACC to VTA‐projecting neurons becomes active during the development of the context‐induced mouse addictive behaviours (McKendrick et al., 2022). In this regard, our discovery of a distinctive pyramidal neuron phenotype with enhanced network processing potential in the ACC may reflect specific changes in the *Snord116del* mouse brain, which increase cortex‐dependent hedonic feeding drive in these mice, and which can be common for other mammalian species, including humans with orthologous genetic mutations. This enhancement in specific brain regions may represent a pathway to obesity in PWS via overactivity of motivation/addiction circuits overriding the homeostatic satiety signals (Rolls, 2012). In support of hedonic feeding hypothesis, a failure to reproduce human PWS obesity phenotype and paradoxical development of HFD‐resistance in *Snord116del* mice might have a revelatory natural explanation (Fig. 7). Chronic repetitive engagement of overactive cortical hedonic feeding circuits may raise stronger compensatory negative feedback response from the neural networks generating sensory‐specific satiety signals (hedonic adaptation) leading to a gradual reduction in the consumption of a repetitively presented palatable food item, while keeping attraction to alternative opportunistic food options (Rolls, 2005, 2012; Schwartz et al., 2017). Predictably, this effect will be more pronounced in animals with a stronger hedonic component of feeding behaviour. Because all previous feeding tests on *Snord116del* mice used a single, non‐alternative palatable food item upon switching from a regular diet to HFD, the progressive development of sensory‐specific satiety in these mice will be stronger compared to controls, rendering a paradoxical HFD‐resistant phenotype. In support of such a mechanism, the switch to HFD suppresses hedonic feeding drive in WT rodents (Arcego et al., 2020; Gazit Shimoni et al., 2025). This effect may diminish the inhibitory power of sensory‐specific satiety on food consumption in control mice that consistently gain weight on HFD, whereas *Snord116del* mice with putative elevated hedonic drive develop an HFD‐resistant phenotype (Ding et al., 2008; Osborne‐Lawrence et al., 2023; Qi et al., 2016) via hedonic adaptation mechanism hypothetically represented in Fig. 7*B*. The likelihood of a similar scenario in human feeding traits was demonstrated in a recent study, in which overweight/obese patients succeeded in losing weight more efficiently if they were able to keep a monotonous diet lacking substantial variety (Hagerman et al., 2026).

Results of our study suggest that previously missed hyperphagic phenotype in *Snord116del* mice might eventually be revealed in specific experimental conditions designed earlier in mouse feeding behavior studies aimed to engage hedonic feeding drive by using ‘cafeteria diet’ regimens that provide a combination of standard chow and a cycling variety of savoury and sweet items to experimental animals (Reichelt et al., 2014). These conditions would mimic human exposure to a high variety of food options that cause overeating in susceptible individuals, dubbed the ‘buffet effect’, in early nutrition studies (Reichelt et al., 2014; Rolls et al., 1981). Our results may give rise to emerging basic and clinical studies aimed at untangling a neurocellular basis of PWS obesity and compulsive behaviours, with regard that early childhood intervention strategies (cognitive behavioural therapy, neuropharmacological or gene therapy approach) might mitigate the development of harmful cortex‐related behavioural anomalies that later during life might result in a permanent pathological shift in homeostatic state or psychiatric status of the patient.

### Future prospective studies

The present work is limited by patch‐clamp electrophysiological approach that uncovered multiple areas for prospective future multidisciplinary studies. *First*, the neurocellular morphological investigation is necessary to verify which parts of the *Snord116del* cortical neurons exhibit hypertrophy manifested by a larger total membrane surface, and whether the dendritic tree overgrowth in these neurons underlies higher basal level of the excitatory input activity. *Second*, more detailed molecular biology study on the regulatory aberrations in the mTOR signalling pathway in *Snord116del* cortical neurons is necessary to identify possible molecular targets for future therapeutic intervention in pathological cases of neuronal hypertrophy or other morphological deformities. *Third*, further electrophysiological investigation is necessary to identify which ion channels contribute to more hyperpolarized *V*_rest_ and delayed firing of APs in *Snord116del* cortical neurons. Properly identified channelopathies can be used for targeted pharmacological or genetic therapy intervention to prevent severe cases of PWS, and they can be informative to develop therapeutic strategies to mitigate negative outcomes of other channelopathies, more often causing an opposite effect of chronic neuronal depolarization. *Fourth*, new prospective feeding behaviour studies should be considered to re‐examine the influence of *Snord116* deficiency on hyperphagia in mice by using various protocols of hedonic contrast paradigm, which in human healthy individuals showed a significant subconscious increase in food consumption (Yeomans et al., 2020). Results of such animal model studies can be used for targeted clinical feeding behaviour tests in PWS patients to clearly identify the impact of hedonic component of hyperphagia and ways to mitigate its effect on lost cognitive control of food consumption, by using appropriate strategies of cognitive behaviour therapy and/or pharmacological treatments.

## Additional information

## Competing interests

None declared.

## Author contributions

Conceptualization, R.K.B.; methodology, V.R.; formal analysis, V.R.; investigation, V.R.; resources, R.K.B.; data curation, V.R.; writing, original draft preparation, V.R.; writing, review and editing, R.K.B.; supervision, R.K.B.; project administration, R.K.B.; funding acquisition, R.K.B. All authors have read and agreed to the published version of the manuscript. All authors approved the final version of the manuscript, agreed to be accountable for all aspects of the work in ensuring that questions related to the accuracy or integrity of any part of the work are appropriately investigated and resolved, and all persons designated as authors qualify for authorship, and all those who qualify for authorship are listed.

## Funding

This work was funded by a grant from the Foundation for Prader–Willi Research.

## Generative AI statement

The Microsoft 365 Copilot UT Southwestern Medical Centre institutional subscription account was used to generate schematic images representing an abstract sagittal section of the human brain, the abstract cartoon representations of the normal and hyperphagic‐driven human subjects, as well as control‐ and high‐fat diet resistant mice, all used in the preparation of Fig. 7.

## Supporting information




Peer Review History

Supporting information

## Data Availability

A comprehensive list of all cells from which the data were collected in this study and their inclusion criteria for calculation of statistical parameters for each measured value are presented in Supplementary Table 1 (Data Inclusion). The extended table containing statistical parameters for all calculated values (included and not included in the article) is presented in Supplementary Table 2 (Data Statistics). The comprehensive tables which include all analyzed differentially expressed genes (DEGs) extracted from original data bases obtained from human PWS samples and Snord116del mouse samples published previously by multiple research laboratories are presented in Supplementary Table 3 (Genomics Databases). All original numerical data sets and Prism GraphPad data files used for statistical analysis of the data will be available from the authors upon reasonable request.
