# Supplementary material for: Altered neural electrophysiological properties in the anterior cingulate cortex in a mouse model of Prader‐Willi syndrome
Source: J Physiol. 2026 Aug 14;604(17):7313–36. doi: 10.1113/JP290255 (PMC13532798; doi:10.1113/JP290255)
Supplement: Supplementary file 1 [file TJP-604-7313-s001.pdf]

The Journal of Physiology

Altered neural electrophysiological properties in the anterior cingulate cortex in a mouse model of Prader-Willi Syndrome

Volodymyr Rybalchenko and Ryan Butler
DOI: 10.1113/JP290255

Corresponding author(s): Ryan Butler (ryan.butler@utsouthwestern.edu)

The following individual(s) involved in review of this submission have agreed to reveal their identity: Shani Stern (Referee #2)

Review Timeline:

Submission Date:	03-Oct-2025
Editorial Decision:	18-Dec-2025
Revision Received:	17-Jun-2026
Editorial Decision:	03-Jul-2026
Revision Received:	05-Jul-2026
Accepted:	14-Jul-2026

Senior Editor: Nathan Schoppa

Reviewing Editor: Jing-Ning Zhu

Transaction Report:

(Note: With the exception of the correction of typographical or spelling errors that could be a source of ambiguity, letters and reports are not edited. Depending on transfer agreements, referee reports obtained elsewhere may or may not be included in this compilation. Referee reports are anonymous unless the Referee chooses to sign their reports.)

Re: JP-RP-2025-290255 "**Paternal *Snord116* deletion alters cortical neuron structure and electrophysiological properties in the mouse anterior cingulate cortex**" by Ryan Butler and Volodymyr Rybalchenko

Dear Dr Butler,

Thank you for submitting your manuscript to The Journal of Physiology. It has been assessed by a Reviewing Editor and by 2 expert referees and we are pleased to tell you that it is potentially acceptable for publication following satisfactory major revision.

Please advise your co-authors of this decision as soon as possible.

The referee reports are copied at the end of this email.

Please address all the points raised and incorporate all requested revisions or explain in your Response to Referees why a change has not been made. We hope you will find the comments helpful and that you will be able to return your revised manuscript within 2 months. If your article is NOT for a Special Issue, you may have 9 months to revise. If you require an extension, please contact journal staff: jp@physoc.org. Please note that this letter does not constitute a guarantee for acceptance of your revised manuscript.

Your revised manuscript should be submitted online using the link in your Author Tasks: Link Not Available. This link is accessible via your account as Corresponding Author; it is not available to your co-authors. If this presents a problem, please contact journal staff (jp@physoc.org). Image files from the previous version are retained on the system. Please ensure you replace or remove any files that are being revised.

If you do not wish to submit a revised version of your manuscript, you must inform our journal staff (jp@physoc.org) or reply to this email to request withdrawal. Please note that a manuscript must be formally withdrawn from the peer review process at one journal before it may be submitted to another journal.

TRANSPARENT PEER REVIEW POLICY: To improve the transparency of its peer review process, The Journal of Physiology publishes online as supporting information the peer review history of all articles accepted for publication. Readers will have access to decision letters, including Editors' comments and referee reports, for each version of the manuscript, as well as any author responses to peer review comments. Referees can decide whether or not they wish to be named on the peer review history document.

ABSTRACT FIGURES: Authors are expected to use The Journal's premium BioRender account to create/redraw their Abstract Figures. Information on how to access this account is here: https://physoc.onlinelibrary.wiley.com/journal/14697793/biorender-access.

This will enable Authors to create and download high-resolution figures. If authors have used the free BioRender service, they can use the instructions provided in the link above to download a high-resolution version suitable for publication.

The link provided should only be used for the purposes of this submission. Authors will be charged for figures created on this account if they are not related to this manuscript submission.

LANGUAGE EDITING AND SUPPORT FOR PUBLICATION: If you would like help with English language editing, or other article preparation support, Wiley Editing Services offers expert help, including English Language Editing, as well as translation, manuscript formatting, and figure formatting at www.wileyauthors.com/eeo/preparation. You can also find resources for Preparing Your Article for general guidance about writing and preparing your manuscript at www.wileyauthors.com/eeo/prepresources.

RIGOUR AND REPRODUCIBILITY: We recommend authors consult The Journal's Rigour and Reproducibility requirements to ensure that their manuscript is suitable for publication: https://physoc.onlinelibrary.wiley.com/pb-assets/hub-assets/physoc/documents/TJP-Rigour-and-Reproducibility-Requirements.pdf

REVISION CHECKLIST:

Check that your Methods section conforms to journal policy: https://jp.msubmit.net/cgi-bin/main.plex?form_type=display_requirements#methods.

Check that data presented conforms to the statistics policy: https://jp.msubmit.net/cgi-bin/main.plex?form_type=display_requirements#statistics.

Upload a full Response to Referees file. To create your 'Response to Referees': copy all the reports, including any comments from the Senior and Reviewing Editors, into a Microsoft Word, or similar, file and respond to each point, using font or background colour to distinguish comments and responses and upload as the required file type.

Please upload two versions of your manuscript text: one with all relevant changes highlighted and one clean version with no changes tracked. The manuscript file should include all tables and figure legends, but each figure/graph should be uploaded as separate, high-resolution files.

You may also upload:

- 'Potential Cover Art' for consideration as the issue's cover image
- Appropriate Supporting Information (Video, audio or data set: see https://jp.msubmit.net/cgi-bin/main.plex?form_type=display_requirements#supp).

We look forward to receiving your revised submission.

If you have any queries, please reply to this email and we will be pleased to advise.

Yours sincerely,

Nathan Schoppa
Senior Editor
The Journal of Physiology

REQUIRED ITEMS

1) - Author photo and profile. First or joint first authors are asked to provide a short biography (no more than 100 words for one author or 150 words in total for joint first authors) and a portrait photograph. These should be uploaded and clearly labelled together in a Word document with the revised version of the manuscript. See Information for Authors for further details.

2) - You must start the Methods section with a paragraph headed Ethical approval (https://jp.msubmit.net/cgi-bin/main.plex?form_type=display_requirements#methods).

Research must comply with The Journal's policies regarding animal experiments (https://physoc.onlinelibrary.wiley.com/hub/animal-experiments) and adherence to these policies must be stated in the manuscript.

Authors should confirm in their Methods section that their experiments were carried out according to the guidelines laid down by their institution's animal welfare committee, including an ethics approval reference number. The Methods section must contain a statement about access to food, water and housing, details of the anaesthetic regime: anaesthetic used, dose and route of administration, and method of killing the experimental animals.

3) - Your manuscript must include a complete Additional Information section, including competing interests; funding; author contributions and acknowledgements.

4) - Please upload separate high-quality figure files via the submission form.

5) - Papers must comply with the Statistics Policy: https://jp.msubmit.net/cgi-bin/main.plex?form_type=display_requirements#statistics.

In summary:

- If n {less than or equal to} 30, all data points must be plotted in the figure in a way that reveals their range and distribution. A bar graph with data points overlaid, a box and whisker plot or a violin plot (preferably with data points included) are acceptable formats.

- If n > 30, then the entire raw dataset must be made available either as supporting information, or hosted on a not-for-profit repository, e.g. FigShare, with access details provided in the manuscript.

- 'n' clearly defined (e.g. x cells from y slices in z animals) in the Methods. Authors should be mindful of pseudoreplication.

- All relevant 'n' values must be clearly stated in the main text, figures and tables.

- The most appropriate summary statistic (e.g. mean or median and standard deviation) must be used. Standard Error of the Mean (SEM) alone is not permitted.

- Exact p values must be stated. Authors must not use 'greater than' or 'less than'. Exact p values must be stated to three significant figures even when 'no statistical significance' is claimed.

6)- Please include an Abstract Figure file and an Abstract Figure legend. An appropriate figure legend, which should not exceed 150 words in length, should be included in the main manuscript file. The Abstract Figure is a piece of artwork designed to give readers an immediate understanding of the research and should summarise the main conclusions. If possible, the image should be easily 'readable' from left to right or top to bottom. It should show the physiological relevance of the manuscript so readers can assess the importance and content of its findings. Abstract Figures should not merely recapitulate other figures in the manuscript. Please try to keep the diagram as simple as possible and without superfluous information that may distract from the main conclusion(s). Abstract Figures must be provided by authors no later than the revised manuscript stage and should be uploaded as a separate file during online submission labelled as File Type 'Abstract Figure'. Please also ensure that you include the figure legend in the main article file. All Abstract Figures should be created using BioRender. Authors should use The Journal's premium BioRender account to export high-resolution images. Details on how to use and access the premium account are included as part of this email.

7) - Please include a full title page as part of your main article (Word) file, which should contain the following: title, authors, affiliations, corresponding author name and contact details, keywords, and running title.

8) - Please ensure that all figures and tables have a title and legend, and that they have been cited within the main article text.

EDITOR COMMENTS

Reviewing Editor:

The authors report electrophysiological changes in pyramidal neurons of the anterior cingulate cortex (ACC) in a mouse model with Snord116 deletion. Both reviewers agree that the manuscript provides novel insight into the role of Snord116 deletion in ACC neuronal electrophysiological properties, but it needs to include evidence of morphological changes and the underlying mTOR signaling pathway. The authors are also encouraged to discuss the link between morphological and electrophysiological changes in ACC neurons and the behavioral deficits observed in Prader-Willi Syndrome, and to provide detailed methods, including anaesthetic and terminal procedures, and the reference number for the ethics approval.

Methods Details:
The authors need to provide details in the Methods section.

Ethics Concerns:
The authors need to provide the reference number of the ethics approval.

Senior Editor:

Thank you for submitting your manuscript to Journal of Physiology. It has been evaluated by two expert referees and a reviewing editor who felt that the study provides valuable insights into the role of Snord116 deletion in altering neuronal properties in the anterior cingulate cortex. The experiments were considered to be generally well-done. A number of concerns however were raised. As outlined in the reviewing editor's report, it was felt that the study would be improved if it provided additional evidence of morphological changes in the neurons and also for the underlying mTOR signaling pathway. In my opinion as senior editor, the authors need not have to address both of these major points with new experiments for the work to be potentially publishable, just one of them. All concerns however should be addressed with either experiments or changes in the analyses and/or text.

Amongst the additional concerns is the need for more detail in the Methods, so that the experiments and analyses are reproducible. Also, there is inadequate information about animal use, including methods of anesthesia, euthanasia, and

terminal procedures. It appears that there are no surgeries, so additional information about that is not needed. The authors provide an IACUC approval number but they should also provide their institution's Animal Welfare Assurance Number.

Please report standard deviations rather than SEMs.

Exact p-values should be reported in the figure legends as well as in the main text.

REFEREE COMMENTS

Referee #1:

This manuscript presents a comprehensive electrophysiological and molecular analysis of pyramidal neurons in the anterior cingulate cortex (ACC) of Snord116-deficient mice, a model for Prader-Willi Syndrome (PWS). The study provides novel insights into how Snord116 loss affects neuronal morphology, intrinsic excitability, and synaptic connectivity, with potential implications for understanding the neural basis of hedonic feeding behavior in PWS.

Major concerns

1.While the electrophysiological data strongly suggest increased dendritic complexity, direct morphological evidence (e.g., dendritic arborization imaging) is lacking. The authors should acknowledge this limitation and consider future studies to validate this assumption.

2.The link between Snord116 deletion and altered gene expression (e.g., mTOR pathway, ion channels) is supported by database mining but remains correlative. Functional validation (e.g., pharmacological or genetic rescue experiments) would strengthen causal claims.

3.Although the authors hypothesize that the observed neuronal changes underlie enhanced hedonic feeding, no direct behavioral data are presented. A discussion of how these specific ACC alterations might translate to feeding behavior in Snord116del mice would be beneficial.

4.The Methods section is overly concise. For all electrophysiological parameters featured in the figures (e.g., action potential threshold, amplitude, etc.), the specific recording protocols and analysis methods should be described in detail within the Methods, rather than being briefly mentioned in the Results. Some statistical details (e.g., exact p-values for all comparisons, correction for multiple comparisons) should be fully reported in the main text or supplementary materials.

Minor concerns

1.It should be specified whether Vrest was measured in I=0 current-clamp mode, and the duration of recording for stabilization should be stated.

2.The protocol for sEPSCs is unclear. It should be stated whether GABA-A receptors were blocked (e.g., with PTX). Furthermore, it should be clarified if the 2-minute analysis period began immediately after membrane rupture or after allowing time for internal solution dialysis and signal stabilization.

3.There is an inconsistency between the Methods (Line 296: 3 ms, 1 nA current) and the Results (Line 364: 10-15 ms, 1 nA). This must be corrected and standardized.

4.The access resistance (Ra) during recordings is not mentioned. The acceptable range or maximum value for Ra should be stated, as this is critical for data quality assessment.

5. In Figure 2, parameters like AP threshold and AP amplitude are typically measured from the same cell. However, the n-values for WT and S116del groups are inconsistent between panels B, C, D, and E. The criteria for including a cell in the analysis for one parameter but not another must be explicitly explained, as this raises concerns about selective data reporting or exclusion. This issue should be checked across all figures.

6. The use of a 50 pA current increment (ΔI=50pA) to measure rheobase is too coarse and not standard practice. A finer step (e.g., 5-10 pA) or a ramp protocol is typically used. Interestingly, the reported mean rheobase values are not multiples of 50 (WT: 139{plus minus}22 pA; S116del: 159{plus minus}26 pA), suggesting a different, unstated analysis method. This protocol and analysis must be described in detail. Given the methodological concern, the conclusion that rheobase is not

different between genotypes is potentially unreliable and requires re-evaluation with a more precise protocol.

7. The identification of a missing subthreshold depolarizing conductance in S116del neurons (active positive to -60 mV) is based solely on I-V curves. The model in Figure 7B speculatively attributes the hyperpolarized Vrest to changes in Na+ and K+ channels. However, no pharmacological interventions (e.g., blockers of persistent Na+ currents or specific K+ channels) were performed to validate the ionic mechanisms underlying the observed differences in Vrest and conductance. Therefore, the model and the associated mechanistic discussion are premature and overly speculative without functional validation.

8. The authors conclude that the more hyperpolarized Vrest in S116del neurons indicates "lower intrinsic membrane excitability" (Line 370). However, Figure 4D1 shows that the firing rate in response to depolarizing current steps is not different between genotypes, which suggests unchanged intrinsic excitability. This apparent contradiction needs to be explicitly addressed and reconciled in the discussion.

Referee #2:

The manuscript presents a comprehensive investigation into the effects of paternal Snord116 deletion on cortical neuron structure and electrophysiological properties in the anterior cingulate cortex (ACC) of a mouse model for Prader-Willi Syndrome (PWS). The study explores the molecular mechanisms underlying neuronal aberrations caused by Snord116 deficiency, which is linked to PWS-a genetic disorder characterized by hyperphagia, obesity, and behavioral abnormalities. Using ex vivo patch-clamp electrophysiology and analysis of genomic databases, the authors provide evidence of altered neuronal connectivity, biophysical properties, and firing patterns in pyramidal neurons (PyNs) of Snord116del mice. The findings suggest that Snord116 deficiency may contribute to enhanced hedonic feeding drive and altered cortical network functionality, offering insights into the pathophysiology of PWS.

Suggestions for Improvement:

1. Include morphological analyses (e.g., dendritic imaging) to confirm the hypothesis of dendritic hypertrophy in Snord116del neurons, alternatively, perform morphological Tracing via rhodamine/ Biocytin/Lucifer yellow dyes of patched cells and MAP2, NeuN and other nneuronal antibodies for immunohistochemical staining of fixed brain slices.

2. analysis of transcriptomic databases indicates an altered expression of genes related to ion channels and mTOR signaling pathways, which may underlie the observed neuronal phenotype. Leading the writers to hypothesized that Increased mTORC2 activity drives the dendritic hypertrophy without changing the soma size of ACC neurons. Yet no past work has clearly and directly demonstrated enhanced activity of the mTORC2 pathway of human or mice in ACC. Therefore, to strengthen this claim it is recommended to preform phospho-substrate Western blots for enhanced mTORC2 activity using protein kinase that phosphorylates targets (Akt S473, SGK1, PKC, etc.).

3. Explore opportunities to validate findings in human PWS patients or human-derived neuronal models to strengthen translational relevance. This may be outside the scope of this study and may require a large body of work. I therefore suggest to at least add a paragrpah in the intro or discussion reviewing related studies or literature reviews.

4. Cgeck ethical requirements for publication in the journal.

END OF COMMENTS

This manuscript presents a comprehensive electrophysiological and molecular analysis of pyramidal neurons in the anterior cingulate cortex (ACC) of Snord116-deficient mice, a model for Prader-Willi Syndrome (PWS). The study provides novel insights into how Snord116 loss affects neuronal morphology, intrinsic excitability, and synaptic connectivity, with potential implications for understanding the neural basis of hedonic feeding behavior in PWS.

Major concerns

1. While the electrophysiological data strongly suggest increased dendritic complexity, direct morphological evidence (e.g., dendritic arborization imaging) is lacking. The authors should acknowledge this limitation and consider future studies to validate this assumption.

2. The link between Snord116 deletion and altered gene expression (e.g., mTOR pathway, ion channels) is supported by database mining but remains correlative. Functional validation (e.g., pharmacological or genetic rescue experiments) would strengthen causal claims.

3. Although the authors hypothesize that the observed neuronal changes underlie enhanced hedonic feeding, no direct behavioral data are presented. A discussion of how these specific ACC alterations might translate to feeding behavior in Snord116del mice would be beneficial.

4. The Methods section is overly concise. For all electrophysiological parameters featured in the figures (e.g., action potential threshold, amplitude, etc.), the specific recording protocols and analysis methods should be described in detail within the Methods, rather than being briefly mentioned in the Results. Some statistical details (e.g., exact p-values for all comparisons, correction for multiple comparisons) should be fully reported in the main text or supplementary materials.

Minor concerns

1. It should be specified whether Vrest was measured in I=0 current-clamp mode, and the duration of recording for stabilization should be stated.
2. The protocol for sEPSCs is unclear. It should be stated whether GABA-A receptors were blocked (e.g., with PTX). Furthermore, it should be clarified if the 2-minute analysis period began immediately after membrane rupture or after allowing time for internal solution dialysis and signal stabilization.
3. There is an inconsistency between the Methods (Line 296: 3 ms, 1 nA current) and the Results (Line 364: 10-15 ms, 1 nA). This must be corrected and standardized.
4.The access resistance (Ra) during recordings is not mentioned. The acceptable range or maximum value for Ra should be stated, as this is critical for data quality assessment.
5. In Figure 2, parameters like AP threshold and AP amplitude are typically measured from the same cell. However, the n-values for WT and S116del groups are inconsistent between panels B, C, D, and E. The criteria for including a cell in the analysis for one

parameter but not another must be explicitly explained, as this raises concerns about selective data reporting or exclusion. This issue should be checked across all figures.

6. The use of a 50 pA current increment (ΔI=50pA) to measure rheobase is too coarse and not standard practice. A finer step (e.g., 5-10 pA) or a ramp protocol is typically used. Interestingly, the reported mean rheobase values are not multiples of 50 (WT: 139±22 pA; S116del: 159±26 pA), suggesting a different, unstated analysis method. This protocol and analysis must be described in detail. Given the methodological concern, the conclusion that rheobase is not different between genotypes is potentially unreliable and requires re-evaluation with a more precise protocol.

7. The identification of a missing subthreshold depolarizing conductance in S116del neurons (active positive to -60 mV) is based solely on I-V curves. The model in Figure 7B speculatively attributes the hyperpolarized Vrest to changes in Na+ and K+ channels. However, no pharmacological interventions (e.g., blockers of persistent Na+ currents or specific K+ channels) were performed to validate the ionic mechanisms underlying the observed differences in Vrest and conductance. Therefore, the model and the associated mechanistic discussion are premature and overly speculative without functional validation.

8. The authors conclude that the more hyperpolarized Vrest in S116del neurons indicates "lower intrinsic membrane excitability" (Line 370). However, Figure 4D1 shows that the firing rate in response to depolarizing current steps is not different between genotypes, which suggests unchanged intrinsic excitability. This apparent contradiction needs to be explicitly addressed and reconciled in the discussion.

This manuscript presents a comprehensive electrophysiological and molecular analysis of pyramidal neurons in the anterior cingulate cortex (ACC) of Snord116-deficient mice, a model for Prader-Willi Syndrome (PWS). The study provides novel insights into how Snord116 loss affects neuronal morphology, intrinsic excitability, and synaptic connectivity, with potential implications for understanding the neural basis of hedonic feeding behavior in PWS.

Major concerns

1. While the electrophysiological data strongly suggest increased dendritic complexity, direct morphological evidence (e.g., dendritic arborization imaging) is lacking. The authors should acknowledge this limitation and consider future studies to validate this assumption.

2. The link between Snord116 deletion and altered gene expression (e.g., mTOR pathway, ion channels) is supported by database mining but remains correlative. Functional validation (e.g., pharmacological or genetic rescue experiments) would strengthen causal claims.

3. Although the authors hypothesize that the observed neuronal changes underlie enhanced hedonic feeding, no direct behavioral data are presented. A discussion of how these specific ACC alterations might translate to feeding behavior in Snord116del mice would be beneficial.

4. The Methods section is overly concise. For all electrophysiological parameters featured in the figures (e.g., action potential threshold, amplitude, etc.), the specific recording protocols and analysis methods should be described in detail within the Methods, rather than being briefly mentioned in the Results. Some statistical details (e.g., exact p-values for all comparisons, correction for multiple comparisons) should be fully reported in the main text or supplementary materials.

Minor concerns

1. It should be specified whether Vrest was measured in I=0 current-clamp mode, and the duration of recording for stabilization should be stated.

2. The protocol for sEPSCs is unclear. It should be stated whether GABA-A receptors were blocked (e.g., with PTX). Furthermore, it should be clarified if the 2-minute analysis period began immediately after membrane rupture or after allowing time for internal solution dialysis and signal stabilization.

3. There is an inconsistency between the Methods (Line 296: 3 ms, 1 nA current) and the Results (Line 364: 10-15 ms, 1 nA). This must be corrected and standardized.

4.The access resistance (Ra) during recordings is not mentioned. The acceptable range or maximum value for Ra should be stated, as this is critical for data quality assessment.

5. In Figure 2, parameters like AP threshold and AP amplitude are typically measured from the same cell. However, the n-values for WT and S116del groups are inconsistent between panels B, C, D, and E. The criteria for including a cell in the analysis for one

parameter but not another must be explicitly explained, as this raises concerns about selective data reporting or exclusion. This issue should be checked across all figures.

6. The use of a 50 pA current increment (ΔI=50pA) to measure rheobase is too coarse and not standard practice. A finer step (e.g., 5-10 pA) or a ramp protocol is typically used. Interestingly, the reported mean rheobase values are not multiples of 50 (WT: 139±22 pA; S116del: 159±26 pA), suggesting a different, unstated analysis method. This protocol and analysis must be described in detail. Given the methodological concern, the conclusion that rheobase is not different between genotypes is potentially unreliable and requires re-evaluation with a more precise protocol.

7. The identification of a missing subthreshold depolarizing conductance in S116del neurons (active positive to -60 mV) is based solely on I-V curves. The model in Figure 7B speculatively attributes the hyperpolarized Vrest to changes in Na+ and K+ channels. However, no pharmacological interventions (e.g., blockers of persistent Na+ currents or specific K+ channels) were performed to validate the ionic mechanisms underlying the observed differences in Vrest and conductance. Therefore, the model and the associated mechanistic discussion are premature and overly speculative without functional validation.

8. The authors conclude that the more hyperpolarized Vrest in S116del neurons indicates "lower intrinsic membrane excitability" (Line 370). However, Figure 4D1 shows that the firing rate in response to depolarizing current steps is not different between genotypes, which suggests unchanged intrinsic excitability. This apparent contradiction needs to be explicitly addressed and reconciled in the discussion.

EDITOR COMMENTS

Reviewing Editor:

The authors report electrophysiological changes in pyramidal neurons of the anterior cingulate cortex (ACC) in a mouse model with Snord116 deletion. Both reviewers agree that the manuscript provides novel insight into the role of Snord116 deletion in ACC neuronal electrophysiological properties, but it needs to include evidence of morphological changes and the underlying mTOR signaling pathway. The authors are also encouraged to discuss the link between morphological and electrophysiological changes in ACC neurons and the behavioral deficits observed in Prader-Willi Syndrome, and to provide detailed methods, including anaesthetic and terminal procedures, and the reference number for the ethics approval.

The authors thank two Reviewers, the Reviewing Editor and the Senior Editor for their comprehensive reviews and valuable suggestions aimed to improve the content and quality of the manuscript. The authors' choice to send the manuscript to the Journal of Physiology was motivated by the historical role of the Journal in validating the cellular patch-clamp electrophysiology as a golden standard and a uniquely rigorous approach in establishing the intracellular ionic homeostasis and dynamics as fundamental mechanisms underlying neuronal communications and animal behaviors. The authors are fully aware that our indirect conclusions on neuronal morphology of Snord116del cortical neurons derived from functional synaptic parameters and genomic database search, prompt ensuing validation via structural morphological study and molecular biology investigation on the involvement of the mTOR signaling pathway controlling neuronal morphology. We planned to conduct these supporting experiments soon enough, only being hampered by sudden loss of financial support from the sponsoring organization. The funding renewal application received both highly enthusiastic and moderately skeptical reviews due to the lack of peer-reviewed published validation of our findings, leaving our lab in a category of prospective recipients. The temporary funding interruption proved consequential: we lost the Snord116del mouse colony maintained on the C57BL/6N background (the most appropriate for studies of the metabolic homeostasis abnormalities, e.g. Mekada & Yoshiki, 2021). We were the last keepers of this mouse colony among our collaborators. Even with funding promptly restored, it would require x10 backcrossing of the commercially available Snord116del(C57BL/6J) on the C57BL/6N background, which takes it beyond realistic timeframe for the experiments suggested by the Reviewers. All other suggestions by the Reviewers and Editors will be addressed point-by-point below.

Methods Details:

The authors need to provide details in the Methods section.

The Methods section was significantly expanded to include all the details required by the Journal of Physiology editorial policies.

Ethics Concerns:
The authors need to provide the reference number of the ethics approval.

The protocol numbers for ethics approval protocols by the host organization were included in the Methods section.

Senior Editor:

Thank you for submitting your manuscript to Journal of Physiology. It has been evaluated by two expert referees and a reviewing editor who felt that the study provides valuable insights into the role of Snord116 deletion in altering neuronal properties in the anterior cingulate cortex. The experiments were considered to be generally well-done. A number of concerns however were raised. As outlined in the reviewing editor's report, it was felt that the study would be improved if it provided additional evidence of morphological changes in the neurons and also for the underlying mTOR signaling pathway. In my opinion as senior editor, the authors need not have to address both of these major points with new experiments for the work to be potentially publishable, just one of them. All concerns however should be addressed with either experiments or changes in the analyses and/or text.

Please consider our explanation above on the Reviewing Editor notes, addressing the inability to conduct the additional experiments within the anticipated framework of the normal manuscript reviewing process. All other concerns by the Reviewers were addressed in the text, figures, hypothesis and conclusions in the revised manuscript.

Amongst the additional concerns is the need for more detail in the Methods, so that the experiments and analyses are reproducible. Also, there is inadequate information about animal use, including methods of anesthesia, euthanasia, and terminal procedures. It appears that there are no surgeries, so additional information about that is not needed. The authors provide an IACUC approval number but they should also provide their institution's Animal Welfare Assurance Number.

The Methods section was significantly expanded to include all the details required by the Journal of Physiology editorial policies.

Please report standard deviations rather than SEMs.

In the revised manuscript, we report SD in the text and all figures, including whiskers on the data plots.

Exact p-values should be reported in the figure legends as well as in the main text.

Exact p-values were added to the figure legends as well as in the main text.

REFEREE COMMENTS

Referee #1:

This manuscript presents a comprehensive electrophysiological and molecular analysis of pyramidal neurons in the anterior cingulate cortex (ACC) of Snord116-deficient mice, a model for Prader-Willi Syndrome (PWS). The study provides novel insights into how Snord116 loss affects neuronal morphology, intrinsic excitability, and synaptic connectivity, with potential implications for understanding the neural basis of hedonic feeding behavior in PWS.

Major concerns

1.While the electrophysiological data strongly suggest increased dendritic complexity, direct morphological evidence (e.g., dendritic arborization imaging) is lacking. The authors should acknowledge this limitation and consider future studies to validate this assumption.

We agree with the reviewers' assessment that direct experiments eliciting specific morphological changes in Snord116del cortical neurons are warranted, which was briefly acknowledged in the original manuscript by suggesting that the future experiments are necessary to confirm our hypothesis. In the reviewed manuscript we additionally accented on this limitation in our study. Our inability to timely conduct the morphological study (as well as other prospective additional experiments) to be included into the manuscript was explained in our response to the Reviewing Editor above.

2.The link between Snord116 deletion and altered gene expression (e.g., mTOR pathway, ion channels) is supported by database mining but remains correlative. Functional validation (e.g., pharmacological or genetic rescue experiments) would strengthen causal claims.

A deeper exploration into the molecular nature of the functional transformation of the Snord116del neurons is a logical next step, which would require more systematic approach than probing at random the contribution of one or two candidate ion conductances, or mTOR pathway elements. We believe

that rescue of Snord116del neuronal phenotype through pharmacological block of one or two ion channels might be informative for professional electrophysiologists, but be potentially confusing for mainstream PWS/obesity research community, and distracting from the main message of the manuscript: hyperphagic phenotype in mammals may potentially result from cortical and motivational neuronal network transformations alongside with generally assumed abnormalities in hypothalamic and metabolic control neurocircuits. However, a separate electrophysiological study addressing the nature of the ionic conductances contributing to abnormally hyperpolarized Vrest and AP spiking patterns would be necessary to unravel prospective directions for pharmacological interventions in PWS patients. A separate study using Snord116del mouse species crossed with mouse species with altered mTOR signaling may be promising to address the role of this signaling pathway in neuronal hypertrophy in Snord116 mice. While we did not perform these additional experiments, we now address the need for them in the discussion.

3.Although the authors hypothesize that the observed neuronal changes underlie enhanced hedonic feeding, no direct behavioral data are presented. A discussion of how these specific ACC alterations might translate to feeding behavior in Snord116del mice would be beneficial.

It is widely accepted that hedonic food perception is subjective and is difficult to evaluate in animals compared to humans, particularly due to general ambiguity in dissecting hedonic component from homeostatic caloric need in rodent feeding behavior tests. There exist several different experimental approaches to study hedonic feeding in rodents, none being accepted as a golden standard though. In the Discussion section of our original manuscript, we mentioned an example of a typical pitfall when reported preference for sucrose over non-caloric saccharin by another PWS model mice PWS^{ICdel}, was declared as evidence of homeostatic rather than hedonic feeding drive (Davies et al., 2015). However, the detailed analysis of the paper revealed that PWS^{ICdel} mice did not show preference for saccharin over water as well, which disqualifies the former as a palatable alternative to sucrose. There exist more rigorous experimental approaches to specifically dissect hedonic component of feeding behavior in mouse models, but those are beyond the scope of the current study (as well as financial limitations). The ACC is a cortical area that encodes palatability of food and generates anticipatory drive of feeding behavior. It is disconnected from direct sensory inputs signaling satiety status that converge in the hypothalamic nuclei. Therefore, neuronal functional changes in the ACC proper would largely influence hedonic drive.

In the absence of funding for our own behavioral studies at this time, we analyzed the totality of published feeding behavioral data on Snord116del mice and discovered clear indication in some publications on pronounced hedonic adaptation (reduced consumption and resultant body weight) compared to controls upon prolong switching to high fat diet (HFT), which may happen only if the hedonic drive itself is stronger than normal in Snord116del mice. Paradoxically, this observation was overlooked in the field (based on homeostatic hypothesis of PWS hyperphagia). Currently, we are looking for funding for our own behavioral studies on Snord116del mice that would focus on hedonic feeding behavior and designed to address existing deficiencies in the experimental design by introducing a wide variety of food items. This approach might override the effects of hedonic adaptation and expose a hyperphagic drive in Snord116del mice. In the meantime, in the revised

manuscript we slightly modified a paragraph discussing a possible link between ACC neuronal alterations and motivational feeding behavior in mammals.

4.The Methods section is overly concise. For all electrophysiological parameters featured in the figures (e.g., action potential threshold, amplitude, etc.), the specific recording protocols and analysis methods should be described in detail within the Methods, rather than being briefly mentioned in the Results. Some statistical details (e.g., exact p-values for all comparisons, correction for multiple comparisons) should be fully reported in the main text or supplementary materials.

We have vastly expanded Methods section in the revised manuscript conforms specific requirements by the JPhysiol and includes all suggestions and requests by the Reviewers.

Minor concerns

1.It should be specified whether Vrest was measured in I=0 current-clamp mode, and the duration of recording for stabilization should be stated.

Added in the Method section. Briefly: I_{hold}*=0 during* V_{rest} *measurements within first 2 min after the membrane rupture;* V_{rest} *typically remained stable during stabilization of ~10 min while measured in current-clamp mode. The initial stabilization time was ~10 min before recordings started. It was to reduce the total duration of the experiment that usually included successive application of several protocols in voltage- and current-clamp modes of recording, and to increase chances that access resistance (Rs) remains stable during the entire experiment.*

2.The protocol for sEPSCs is unclear. It should be stated whether GABA-A receptors were blocked (e.g., with PTX). Furthermore, it should be clarified if the 2-minute analysis period began immediately after membrane rupture or after allowing time for internal solution dialysis and signal stabilization.

Added in the Method section. Briefly: The pipette solution composition was designed to keep the Nernst equilibrium potential for Cl- anions at ~ -70 mV. We recorded sEPSCs at Vh=-70 mV in voltage-clamp mode, which efficiently nullified GABA-A receptor mediated Cl- currents during recordings. We purposely did not block GABA-A receptors, because after switching to the current-clamp mode, we intended to keep the neuron in the most natural environmental state with unchanged Vrest and AP firing patterns. Blocking the tonic contribution of the background GABAA receptor synaptic inputs would inevitably skew both measured metrics. We started recording 2 min contiguous traces of the sEPSCs after >10 min of pipette solution equilibration to make sure that GABAA receptor-mediated sIPSCs are nullified.

3.There is an inconsistency between the Methods (Line 296: 3 ms, 1 nA current) and the Results (Line 364: 10-15 ms, 1 nA). This must be corrected and standardized.

We are grateful for expressing this particular concern. This inconsistency originated from a mistakenly produced wrong number shown over the time-scale bar in Fig. 2A. Instead of 2 ms (corrected in a revised manuscript) a wrong tag of 20 ms was shown in the original Fig. 2A, resulting in unverified visual assessment and further description of the duration of the 1 nA stimulation pulse in the Results section, being ~10 times longer than real values. Corrected in the Results section of the revised manuscript.

4.The access resistance (Ra) during recordings is not mentioned. The acceptable range or maximum value for Ra should be stated, as this is critical for data quality assessment.

Added in the Method section. The access resistance (alternatively the series resistance R_s) typically ranged within 15-30 MΩ, and few cells with Rs~35 MΩ. were included. The possible R_s skewing effect on the recorded parameters was minimized by routinely engaging a standard electronic R_s compensation feedback circuitry embedded in the Clampex 10.2 recording software interface. R_s was periodically verified during the experiment, and collected data were rejected if- or after the R_s exceeded the initial value by 25%.

5. In Figure 2, parameters like AP threshold and AP amplitude are typically measured from the same cell. However, the n-values for WT and S116del groups are inconsistent between panels B, C, D, and E. The criteria for including a cell in the analysis for one parameter but not another must be explicitly explained, as this raises concerns about selective data reporting or exclusion. This issue should be checked across all figures.

To address this 'minor' concern, the comprehensive overhaul of all the data and experiments' log files was accomplished, which took a major portion of the entire manuscript revision process. The experimental data that formed the original manuscript were progressively accumulated in the statistical pool over extended period of time, and we at first experienced impediments while tracking and verifying the cell inclusion criteria for each particular experiment. To mitigate the problem, we created a separate Excel spread sheet file in which each cell included in the original study is listed alongside the Figure numbers, where the data from this cell was included into the analysis. This File also includes additional notes and observations from log files created at the time of the experiment. This approach elucidated that primary variability in the number of cells included in one or another experiment originated from a few major sources: i) the sequence and number of applied experimental protocols (both voltage- and current clamp modes) varied from cell to cell (e.g. to collect additional data for the initially underrepresented data sets); ii) some planned protocols could not be applied to the cell if the experiment stopped prematurely; iii) experimental mice (either Snord116del or controls) were not always available when needed, resulting in non-even genotype representation. Typical

*examples of unexpected, but justified exclusion or missing data points were as follows: N for C_m>R_m by 2 because C_m was reliably measured in two loosely sealed cells with compromised R_m measurements; some cells showing convincingly hyperpolarized V_{rest} produced none- or only one AP in leu of bursts of APs upon depolarizing current steps; the unstable current baseline prevented reliable recording of sEPSCs but not macroscopic currents in voltage-clamp mode in some cells, etc. While compiling the data inclusion Excel file, we discovered that some data points were overlooked or not included in the original data pool. E.g., V_{rest} values for the number of cells tested subsequently only in voltage-clamp mode, were not included in the original pool of data but added in the revised manuscript. Besides, all previously discarded data obtained from three cells with V_{rest} values of -49, -46 and -45 mV were added to the total pool after revelation that normalized membrane conductance (G_m/C_m) for these cells was within 0.4*SD from the mean values for their genotype group, meaning that depolarized subthreshold resting-state occurred naturally, and nor as a result of poor seal or leaky membrane. Besides, we excluded two data points from the AP_{thresh} data set (Fig. 2C) included in the original manuscript, because they were obtained from the cells hyperpolarized at rest only due to the injection of hyperpolarizing current. The "Snord116 Data Inclusion" Excel file is added to the additional materials accompanying the revised manuscript to assure full transparency of the study. The explained above, minor revision of the data sets in the revised manuscript did not change noticeably the statistically significant differences for measured parameters between genotypes in all but two data sets: the height of the voltage barrier to fire an AP (V_{thr} - V_{rest}) measured in individual cells (Fig. 2D) fell short of the significant difference criterium (Mann-Whitney test p=0.14); and in reverse, the small separation between curves for "runaway firing frequency" (Fig. 4D1) became more regular between two genotypes to reach formal statistically significant difference criterium (Wilcoxon paired test, p= 0.018), although very small absolute difference between means renders this difference of little physiological importance.*

6. The use of a 50 pA current increment (ΔI=50pA) to measure rheobase is too coarse and not standard practice. A finer step (e.g., 5-10 pA) or a ramp protocol is typically used. Interestingly, the reported mean rheobase values are not multiples of 50 (WT: 139{plus minus}22 pA; S116del: 159{plus minus}26 pA), suggesting a different, unstated analysis method. This protocol and analysis must be described in detail. Given the methodological concern, the conclusion that rheobase is not different between genotypes is potentially unreliable and requires re-evaluation with a more precise protocol.

Initially, a rheobase was not among the priority parameters for which the application of a separate protocol was planned. The application of a conventional protocol with small ΔI=10pA steps followed by at least 10 s interpulse interval would "bite" quite a significant portion of time out of the entire duration of experiment (up to 40 steps for some cells), while successive application of multiple protocols to the same cell was planned in advance. (The application of the ramp protocol was not considered applicable due to the concerns that initial slow component of the ramp might produce partial inactivation of the Na+ channels and introduce unaccounted artificial positive shift to the measured rheobase value). After releasing later that rheobase might be informative in understanding the differences in subthreshold voltage sensitivity between two genotypes, we decided to calculate the chord values for rheobase, extracted from ΔI=50pA 500ms current steps protocols that we used

to study the patterns of AP series. We applied the formula $I_{rhe}=I_{last}+10*(6-n)$, where I_{last} is the last step that produced no APs, and n is the number of APs generated upon the next resultative step (n=5 was set in a few cells with more than 5 APs generated upon the first resultative step). We added the description of the rheobase calculation formula in the Method section.

7. The identification of a missing subthreshold depolarizing conductance in S116del neurons (active positive to -60 mV) is based solely on I-V curves. The model in Figure 7B speculatively attributes the hyperpolarized Vrest to changes in Na+ and K+ channels. However, no pharmacological interventions (e.g., blockers of persistent Na+ currents or specific K+ channels) were performed to validate the ionic mechanisms underlying the observed differences in Vrest and conductance. Therefore, the model and the associated mechanistic discussion are premature and overly speculative without functional validation.

Unfortunately, due to the reasons explained above, and while waiting for the renewal of financial support, we cannot perform additional suggested experiments (within limited timescale for the revision process) to validate molecular aspects of our hypothesis outlined in Fig. 7B. Even though we presented the original Fig. 7 in the main text as hypothetical, we accentuated in revised version a point that this mechanism is just a likely scenario by putting a question mark over this part of the figure. We also removed a schematic representation of the extended dendritic tree lacking direct experimental support in this manuscript. We keep Fig. 7 as an important part of the manuscript due to the accent in the figure on a possible hedonic nature of hyperphagia in humans (since ACC is a part of reward search network, not a homeostatic state signaling system), while drawing attention to limitations of previous feeding behavioral tests to uncover potential hedonic feeding overdrive in Snord116del mice (presentation of non-alternative high-fat diet chaw resulting in no weight gain).

8. The authors conclude that the more hyperpolarized Vrest in S116del neurons indicates "lower intrinsic membrane excitability" (Line 370). However, Figure 4D1 shows that the firing rate in response to depolarizing current steps is not different between genotypes, which suggests unchanged intrinsic excitability. This apparent contradiction needs to be explicitly addressed and reconciled in the discussion.

Thanks for expressing this concern. We admit that expression "lower intrinsic membrane excitability" in a particular context of description of results in Fig. 2B-D is rather confusing. We tried to communicate a concept that Snord116del neurons in the absence of excitatory input will be less spontaneously active compared to controls due to more hyperpolarized V_{rest}. This part of the Results section was rewritten to clarify our interpretation of the results in Fig. 2, and additionally to incorporate revised data in Fig. 2D for a parameter (V_{thr} - V_{rest}) measured in individual cells, which changed a status from 'significantly different' between two genotypes to 'showing tendency to be higher in Snord116del neurons' after full revision of all data sets presented in all Figures (see above). We also admit that there is no conventional definition for neuronal intrinsic excitability. Therefore, we used a borrowed term "runaway firing frequency" to describe the data in Fig. 4D1. This is to recognize that

maximal neuron firing frequency in response to injection of high suprathreshold current steps of ~500 ms duration, often referred to as 'neuronal excitability', is a non-physiological descriptor of mere neuronal ability to produce high frequency regenerative APs in artificial experimental conditions of high injected current, which never occur in real physiological conditions. Instead, in following Fig. 5 and 6 we explore the neuronal ability to generate specific series of APs (i.e. 'physiological excitability') by applying minimal-to-moderate current pulses modeling physiological levels of combined dendritic excitatory input. We tried to make this distinction clearer in the revised manuscript. Among other considerations of the 'neuronal excitability' in revised manuscript, we found that after addition of the data from three previously discarded cells, the full sets of data points for runaway firing frequency (Fig. 4D1) became slightly more separated between two genotypes to reach a formal significant difference criteria (Wilcoxon paired test, p= 0.018), although being of little potential physiological importance (mentioned above in response to other 'Minor concern').

Referee #2:

The manuscript presents a comprehensive investigation into the effects of paternal Snord116 deletion on cortical neuron structure and electrophysiological properties in the anterior cingulate cortex (ACC) of a mouse model for Prader-Willi Syndrome (PWS). The study explores the molecular mechanisms underlying neuronal aberrations caused by Snord116 deficiency, which is linked to PWS-a genetic disorder characterized by hyperphagia, obesity, and behavioral abnormalities. Using ex vivo patch-clamp electrophysiology and analysis of genomic databases, the authors provide evidence of altered neuronal connectivity, biophysical properties, and firing patterns in pyramidal neurons (PyNs) of Snord116del mice. The findings suggest that Snord116 deficiency may contribute to enhanced hedonic feeding drive and altered cortical network functionality, offering insights into the pathophysiology of PWS.

Suggestions for Improvement:

1. Include morphological analyses (e.g., dendritic imaging) to confirm the hypothesis of dendritic hypertrophy in Snord116del neurons, alternatively, perform morphological Tracing via rhodamine/ Biocytin/Lucifer yellow dyes of patched cells and MAP2, NeuN and other neuronal antibodies for immunohistochemical staining of fixed brain slices.

We understand that morphological analysis is a next logical step to verify experimentally our preliminary conclusion on Snord116del cortical neuron hypertrophy based on our electrophysiological data and RNAseq data mining. Unfortunately, in our response above to the Reviewing Editor, we explained that we are not capable to conduct any additional experiments at this time due to a temporary loss of financial support for our research project, resulting in a concomitant loss of the Snord116del(C57BL/6J) mouse line. The renewal of financial support is highly dependent on the

successful publication of our electrophysiological study in an appropriate highly recognized peer-reviewed journal such as JPysiol.

2. analysis of transcriptomic databases indicates an altered expression of genes related to ion channels and mTOR signaling pathways, which may underlie the observed neuronal phenotype. Leading the writers to hypothesized that Increased mTORC2 activity drives the dendritic hypertrophy without changing the soma size of ACC neurons. Yet no past work has clearly and directly demonstrated enhanced activity of the mTORC2 pathway of human or mice in ACC. Therefore, to strengthen this claim it is recommended to preform phospho-substrate Western blots for enhanced mTORC2 activity using protein kinase that phosphorylates targets (Akt S473, SGK1, PKC, etc.).

We would be happy to conduct this valuable series of experiments soon after the financial support of our research project is restored.

3. Explore opportunities to validate findings in human PWS patients or human-derived neuronal models to strengthen translational relevance. This may be outside the scope of this study and may require a large body of work. I therefore suggest to at least add a paragrpah in the intro or discussion reviewing related studies or literature reviews.

In our Discussion, we devoted a separate subsection to analyze relevant links between a known role of ACC in human hedonic and compulsive feeding, and reported hyperactivity of the ACC in PWS patients revealed through fMRI investigations. One straightforward approach for translational studies based on our research would be a targeted human hedonic feeding behavior study recruiting the PWS patients with confirmed Snord116 deficiency. Such a study would be realistic in future, but we plan first to design, acquire funding, and conduct Snord116del mouse hedonic feeding behavior study to validate the next step for prospective clinical human study. We discussed this prospective for future studies at the end of the Discussion section in the original manuscript. In the revised manuscript, we separated this, and other valuable suggestions by the Reviewers of this manuscript, into a separate 'Future Prospective Studies' subsection at the end of the manuscript.

With regard of the human-derived neuronal models, in our manuscript we mentioned a recent genetic study on PWS using neuronal cells derived from human embryonic stem cells (hESC) with targeted Snord116 deletions (Gilmore et al., 2024). Interestingly, the authors reported among ~ 40 most consistently dysregulated genes the FGF13, which is a positive regulator of Na+ voltage-dependent ion channels that initiate- and drive AP firing by neuronal soma. Contrary to results of our data mining, reporting significant upregulation of members of same molecular family (FGF12, FEG14) in Snord116del mouse cortical tissue, Gilmore et al. reported significant downregulation of FGF13 in hESC derived neuronal cells. The discrepancy becomes easily explained after revelation that hESC-derived "neurons" in their study are not electrically excitable (i.e. unable to generate AP), manifesting failure to express functional Na+ voltage-dependent ion channels. Alternatively, the oppositely (i.e. negatively) regulated FGF13 in Snord116del hESCs render the endogenous Na+ ion channels non-

functional, questioning validity of the Snord116del hESC-derived "neuronal" model to study neuronal excitability in PWS.

4. Check ethical requirements for publication in the journal.

The Methods section of the revised manuscript was significantly expanded to accommodate multiple suggestions by the Reviewers and Editors, including additional previously missing information on ethical requirements.

END OF COMMENTS

Dear Dr Butler,

Re: JP-RP-2026-290255R1 "**Paternal *Snord116* deletion alters cortical neuron structure and electrophysiological properties in the mouse anterior cingulate cortex**" by Volodymyr Rybalchenko and Ryan Butler

Thank you for submitting your manuscript to The Journal of Physiology. It has been assessed by a Reviewing Editor and by 2 expert referees and we are pleased to tell you that it is acceptable for publication following satisfactory revision.

Please share this decision with your co-authors as soon as possible.

The referee reports are copied at the end of this email.

Please address all the points raised and incorporate all requested revisions or explain in your Response to Referees why a change has not been made. We hope you will find the comments helpful and that you will be able to return your revised manuscript within 4 weeks. If you require longer than this, please contact journal staff: jp@physoc.org.

Your revised manuscript should be submitted online using the link in your Author Tasks Link Not Available. This link is accessible via your account as Corresponding Author; it is not available to your co-authors. If this presents a problem, please contact journal staff (jp@physoc.org). Image files from the previous version are retained on the system. Please ensure you replace or remove any files that are being revised.

If you do not wish to submit a revised version of your manuscript, you must inform our journal staff (jp@physoc.org) or reply to this email to request withdrawal. Please note that a manuscript must be formally withdrawn from the peer review process at one journal before it may be submitted to another journal.

TRANSPARENT PEER REVIEW POLICY: To improve the transparency of its peer review process The Journal of Physiology publishes online, as supporting information, the peer review history of all articles accepted for publication. Readers will have access to decision letters, including Editors' comments and referee reports, for each version of the manuscript, as well as any author responses to peer review comments. Referees can decide whether or not they wish to be named on the peer review history document.

ABSTRACT FIGURES: Authors are expected to use The Journal's premium BioRender account to create/redraw their Abstract Figures. Information on how to access this account is here: https://physoc.onlinelibrary.wiley.com/journal/14697793/biorender-access.

This will enable Authors to create and download high-resolution figures. If authors have used the free BioRender service, they can use the instructions provided in the link above to download a high-resolution version suitable for publication.

The link provided should only be used for the purposes of this submission. Authors will be charged for figures created on this account if they are not related to this manuscript submission.

LANGUAGE EDITING AND SUPPORT FOR PUBLICATION: If you would like help with English language editing, or other article preparation support, Wiley Editing Services offers expert help, including English Language Editing, as well as translation, manuscript formatting, and figure formatting at www.wileyauthors.com/eeo/preparation. You can also find resources for Preparing Your Article for general guidance about writing and preparing your manuscript at www.wileyauthors.com/eeo/prepresources.

RIGOUR AND REPRODUCIBILITY: We recommend authors consult The Journal's Rigour and Reproducibility requirements to ensure that their manuscript is suitable for publication: https://physoc.onlinelibrary.wiley.com/pb-assets/hub-assets/physoc/documents/TJP-Rigour-and-Reproducibility-Requirements.pdf

REVISION CHECKLIST:

Check that your Methods section conforms to journal policy: https://jp.msubmit.net/cgi-bin/main.plex?form_type=display_requirements#methods

Check that data presented conforms to the statistics policy: https://jp.msubmit.net/cgi-bin/main.plex?form_type=display_requirements#statistics

Upload a full Response to Referees file. To create your 'Response to Referees' copy all the reports, including any comments from the Senior and Reviewing Editors, into a Microsoft Word, or similar, file and respond to each point, using font or background colour to distinguish comments and responses and upload as the required file type.

Please upload two versions of your manuscript text: one with all relevant changes highlighted and one clean version with no changes tracked. The manuscript file should include all tables and figure legends, but each figure/graph should be uploaded as separate, high-resolution files. The journal is now integrated with Wiley's Image Checking service. For further details,

see: https://www.wiley.com/en-us/network/publishing/research-publishing/trending-stories/upholding-image-integrity-wileys-image-screening-service

You may also upload:

- 'Potential Cover Art' for consideration as the issue's cover image
- Appropriate Supporting Information (Video, audio or data set: see https://jp.msubmit.net/cgi-bin/main.plex?form_type=display_requirements#supp).

We look forward to receiving your revised submission.

If you have any queries, please reply to this email and we will be pleased to advise.

Yours sincerely,

Nathan Schoppa
Senior Editor
The Journal of Physiology

EDITOR COMMENTS

Reviewing Editor:

Thank you for your detailed responses to the reviewers' and my comments. Both reviewers are very satisfied with the revisions. I have no further questions.

Senior Editor:

Thank you for submitting your revised manuscript to Journal of Physiology. It has been reviewed by the two external reviewers of your original manuscript and a reviewing editor, and it was felt that the revision adequately addresses the prior concerns that were raised. It was noted that, while the authors were not able to include additional morphological analyses of neuronal structure, the revisions were substantial and the work makes a substantial contribution towards the understanding of the neural basis of Prader-Willi Syndrome.

One additional point that I would like to make as senior editor is that I would strongly encourage the authors to alter the title of their study, given the absence of direct measurements of cortical neuron structure. The present title leaves readers expecting an analysis of neuronal structure. An appropriate title might be "Paternal Snord116 deletion alters cortical neuron electrophysiological properties in the mouse anterior cingulate cortex". An alternative title that would also hint at the potential clinical significance of the work (useful for a general audience of JP readers and thus preferred by me) would be something like "Altered neural electrophysiological properties in the anterior cingulate cortex in a mouse model of Prader-Willi Syndrome".

REFEREE COMMENTS

Referee #1:

The authors have adequately addressed my concerns. I appreciate their patience and thorough responses.

Referee #2:

I am satisfied with the revision and the paper is ready for publication.

END OF COMMENTS

Reviewing Editor:

Thank you for your detailed responses to the reviewers' and my comments. Both reviewers are very satisfied with the revisions. I have no further questions.

Senior Editor:

Thank you for submitting your revised manuscript to Journal of Physiology. It has been reviewed by the two external reviewers of your original manuscript and a reviewing editor, and it was felt that the revision adequately addresses the prior concerns that were raised. It was noted that, while the authors were not able to include additional morphological analyses of neuronal structure, the revisions were substantial and the work makes a substantial contribution towards the understanding of the neural basis of Prader-Willi Syndrome.

One additional point that I would like to make as senior editor is that I would strongly encourage the authors to alter the title of their study, given the absence of direct measurements of cortical neuron structure. The present title leaves readers expecting an analysis of neuronal structure. An appropriate title might be "Paternal Snord116 deletion alters cortical neuron electrophysiological properties in the mouse anterior cingulate cortex". An alternative title that would also hint at the potential clinical significance of the work (useful for a general audience of JP readers and thus preferred by me) would be something like "Altered neural electrophysiological properties in the anterior cingulate cortex in a mouse model of Prader-Willi Syndrome".

We deeply appreciate the time and effort of the editors and reviewers of our manuscript and are delighted that they feel it is suitable for publication. Per the senior editors' request, we have changed the title to, "Altered neural electrophysiological properties in the anterior cingulate cortex in a mouse model of Prader-Willi Syndrome."

REFEREE COMMENTS

Referee #1:

The authors have adequately addressed my concerns. I appreciate their patience and thorough responses.

Referee #2:

I am satisfied with the revision and the paper is ready for publication.

2nd Revision - Editorial Decision **14-Jul-2026**

Dear Dr Butler,

Re: JP-RP-2026-290255R2 "**Altered neural electrophysiological properties in the anterior cingulate cortex in a mouse model of Prader-Willi Syndrome**" by Volodymyr Rybalchenko and Ryan Butler

We are pleased to tell you that your paper has been accepted for publication in The Journal of Physiology.

The last Word (or similar) version of the manuscript provided will be used by the Production Editor to prepare your proof. When this is ready you will receive an email containing a link to Wiley's Online Proofing System. The proof should be thoroughly checked and corrected as promptly as possible.

Authors should note that it is too late at this point to offer corrections prior to proofreading. Major corrections at proof stage, such as changes to figures, will be referred to the Editors for approval before they can be incorporated. Only minor changes, such as to style and consistency, should be made at proof stage. Changes that need to be made after proof stage will usually require a formal correction notice.

All queries at proof stage should be sent to: TJP@wiley.com.

Following copyediting and typesetting the accepted version will be published online.

Yours sincerely,

Nathan Schoppa
Senior Editor
The Journal of Physiology

IMPORTANT POINTS TO NOTE FOLLOWING ACCEPTANCE OF YOUR PAPER:

• IMPORTANT NOTICE ABOUT OPEN ACCESS: To assist authors whose funding agencies mandate immediate public access to published research findings, The Journal of Physiology allows authors to pay an Open Access (OA) fee to have their papers made freely available immediately on publication.

The Corresponding Author will receive an email from Wiley with details on how to register or log in to Wiley Authors where you will be able to place an order.

• You can check if your funder or institution has a Wiley Open Access Account here:
https://authors.wiley.com/author-resources/Journal-Authors/open-access/author-compliance-tool.html

MEMBER DISCOUNT: Corresponding authors who are Members of the Physiological Society are eligible for a 20% discount on the cost of the Article Processing Charge (APC). To claim this discount, the Corresponding Author will need to log in to the Physiological Society Membership portal and retrieve the discount code (https://www.physoc.org/journals-and-media/journals/journal-discount-for-members/). This code should then be quoted in the billing section of Wiley Authors when selecting the open access option.

• TRANSPARENT PEER REVIEW POLICY: To improve the transparency of its peer review process, The Journal of Physiology publishes online as supporting information the peer review history of all articles accepted for publication. Readers will have access to decision letters, including Editors' comments and referee reports, for each version of the manuscript as well as any author responses to peer review comments. Referees can decide whether or not they wish to be named on the peer review history document.

• You can help your research get the attention it deserves! Check out Wiley's free Promotion Guide for best-practice recommendations for promoting your work at: www.wileyauthors.com/eeo/guide. You can learn more about Wiley Editing Services which offers professional video, design, and writing services to create shareable video abstracts, infographics, conference posters, lay summaries, and research news stories for your research at: www.wileyauthors.com/eeo/promotion.

• If you would like to receive our 'Research Roundup', a monthly newsletter highlighting the cutting-edge research published in The Physiological Society's family of journals (The Journal of Physiology, Experimental Physiology, Physiological Reports, The Journal of Nutritional Physiology and The Journal of Precision Medicine: Health and Disease), please click this link, fill in your name and email address and select 'Research Roundup':
https://www.physoc.org/journals-and-media/membernews

EDITOR COMMENTS

Senior Editor:

We appreciate that you have changed the title of your manuscript. It is now acceptable for publication. Congratulations on the nice study!
